# Transitions from Single- to Multi-Locus Processes during Speciation with Gene Flow

**DOI:** 10.3390/genes9060274

**Published:** 2018-05-24

**Authors:** Martin P. Schilling, Sean P. Mullen, Marcus Kronforst, Rebecca J. Safran, Patrik Nosil, Jeffrey L. Feder, Zachariah Gompert, Samuel M. Flaxman

**Affiliations:** 1 Department of Ecology and Evolutionary Biology, University of Colorado, Boulder, CO 80309, USA; rebecca.safran@colorado.edu (R.J.S.); Samuel.Flaxman@colorado.edu (S.M.F.); 2 Department of Biology, Boston University, Boston, MA 02215, USA; smullen@bu.edu; 3 Department of Ecology & Evolution, University of Chicago, Chicago, IL 60637, USA; mkronforst@uchicago.edu; 4 Department of Biology & Ecology Center, Utah State University, Logan, UT 84322, USA; p.nosil@sheffield.ac.uk (P.N.); zach.gompert@usu.edu (Z.G.); 5 Department of Biological Sciences, University of Notre Dame, South Bend, IN 46556, USA; Jeffrey.L.Feder.2@nd.edu

**Keywords:** gene flow, sympatry, parapatry, simulation model, population genomics, *Heliconius*, coupling, nonlinear transitions

## Abstract

During speciation-with-gene-flow, a transition from single-locus to multi-locus processes can occur, as strong coupling of multiple loci creates a barrier to gene flow. Testing predictions about such transitions with empirical data requires building upon past theoretical work and the continued development of quantitative approaches. We simulated genomes under several evolutionary scenarios of gene flow and divergent selection, extending previous work with the additions of neutral sites and coupling statistics. We used these simulations to investigate, in a preliminary way, if and how selected and neutral sites differ in the conditions they require for transitions during speciation. For the parameter combinations we explored, as the per-locus strength of selection grew and/or migration decreased, it became easier for selected sites to show divergence—and thus to rise in linkage disequilibrium (LD) with each other as a statistical consequence—farther in advance of the conditions under which neutral sites could diverge. Indeed, even very low rates of effective gene flow were sufficient to prevent differentiation at neutral sites. However, once strong enough, coupling among selected sites eventually reduced gene flow at neutral sites as well. To explore whether similar transitions might be detectable in empirical data, we used published genome resequencing data from three taxa of *Heliconius* butterflies. We found that fixation index ($F_{ST}$) outliers and allele-frequency outliers exhibited stronger patterns of within-deme LD than the genomic background, as expected. The statistical characteristics of within-deme LD—likely indicative of the strength of coupling of barrier loci—varied between chromosomes and taxonomic comparisons. Qualitatively, the patterns we observed in the empirical data and in our simulations suggest that selection drives rapid genome-wide transitions to multi-locus coupling, illustrating how divergence and gene flow interact along the speciation continuum.

## 1. Introduction

Understanding the genetic basis of speciation—long a central goal of evolutionary biology—has been greatly advanced by high-throughput sequencing (HTS) methods. High-quality datasets from a wealth of empirical studies of speciation-in-action now abound, and we can now point to many excellent examples demonstrating that combinations of factors—selection, drift, ecology, geography, hybridization, recombination, and more—shape divergence and reproductive isolation (e.g., [1,2]). However, the current abundance of data also underscores the vast gulf that still often exists between “having the data” and “having the answers.” Signs of varied evolutionary processes can sometimes be unambiguously detected, but predicting and testing for the patterns of aggregate, genome-wide processes along the speciation continuum (i.e., at varying points of divergence and differentiation) remains challenging. Therefore, additional work is needed to expand the scale of prediction and analysis from sets of “speciation genes” to genome-wide statistical patterns.

As Butlin and Smadja [3] recently highlighted, theory based upon coupling is a promising foundation for this work. In this context, “coupling” refers to any process that combines barrier effects from multiple loci, leading to strengthening of the overall barrier to gene flow [3]. Coupling involves alleles at different loci becoming statistically non-independent with respect to their evolutionary dynamics. For example, two loci that are subject to divergent selection may together, when coupled, reach allele frequency differences (AFD) between demes that are larger than either allele would reach at the corresponding uncoupled, single-locus migration-selection balance. In general, the potential for loci to be coupled is strongly dependent upon the relative strengths of selection and recombination ([4], more on this below). It is also important to note that the notion of coupling as defined here can include but is not limited to allele frequency clines becoming coincident in space. Butlin and Smadja [3] pointed out that “coupling” is not synonymous with “linkage disequilibrium” (LD). Here, we focus on barriers to gene flow arising from “two-allele effects” (sensu [5]), and for this reason coupling and LD go hand in hand in the simulation results we present below.

Multi-locus processes involved in speciation have been studied by theoreticians for decades [1,6], providing an excellent foundation to understanding the build-up and maintenance of differentiation under selection, gene flow, and genetic drift [3,4,6,7,8,9,10,11]. A number of theoretical studies have also cast light upon the roles of linkage and genomic architecture in speciation [12,13,14,15,16,17,18]. Much of this work has emphasized the parameter space—combinations of selection strength, migration rates, recombination rates, numbers of loci, etc.—in which loci would become coupled with one another (e.g., [4,7,9,10,19]).

Recent work has additionally emphasized the temporal properties of the emergence of coupling, and indicates that the build-up of divergence can be strongly nonlinear in time [13,18,20,21,22,23]. Specifically, these studies suggest that many alleles with individually small effects may rapidly (in evolutionary terms) transition from an uncoupled to a coupled state, a process in which highly divergently adapted multi-locus genotypes “congeal” out of what was previously a well-mixed gene pool [18]. However, the latter work did not incorporate neutral sites and thus was silent about their dynamics.

Bürger and Akerman [24] and Akerman and Bürger [25] explored the joint effect of recombination and gene flow on a single neutral site in close proximity to one selected site or between two weakly selected sites, respectively. It was found that high LD and strong barriers to gene flow can arise at such neutral sites, increasing neutral differentiation between two populations. Additionally, Aeschbacher and Bürger [26] estimated the distribution of allele frequencies, population divergence, as well as coalescence rates at a single neutral mutation close to an arbitrary number of selected sites. Similar to Bürger and Akerman [24] and Akerman and Bürger [25], it was found that the distance of a neutral locus would have to be relatively small for neutral divergence to increase greatly (see also [27,28,29]). However, Bürger and Akerman [24], Akerman and Bürger [25], Aeschbacher and Bürger [26] explored whether islands of speciation would emerge using a single mutation, without allowing for mutations at multiple loci to arise over long periods of evolutionary time and interact via linkage. Here, we present simulations in which millions of mutations—selected and neutral, and dispersed all over a genome of multiple chromosomes—arise during each simulation run.

Southcott and Kronforst [30] used forward-time simulations that included multiple neutral sites in a large (100 kb) region of the genome containing a single site under selection. Their work suggested that the genomic patterns produced by neutral and non-neutral processes may not be easily distinguishable. This raises important questions for exploratory analyses: Are there ways to reliably distinguish barrier loci under selection from neutral genomic background? Can aggregate, genome-wide statistical patterns offer insights about neutral and non-neutral processes? What, if any, patterns produced by neutral versus non-neutral processes are robust and detectable?

Toward this end, we investigated the temporal dynamics of coupling in two-deme simulations under divergent selection and gene flow, spanning a range of migrants per generation between 0.1 and 500. We built on a previous model (“*bu2s*”: [18]) that considered millions of de novo mutations leading to the build-up of large numbers (100 s) of sites under selection and extended that model to incorporate neutral sites. Following the results about the difficulty of using traditional summary statistics to distinguish non-neutral and neutral evolutionary processes [30], we used results from the model to compare the potential power of multiple population genetic statistics to differentiate between neutral and selected sites. We also computed coupling statistics derived from multi-locus cline theory [4,9] to quantitatively describe transitions from single- to multi-locus processes during speciation with gene flow. Consistent with previous work, selected sites frequently transitioned from low to high coupling between loci, and we now provide quantitative predictions about the time and parameter lag between the transition seen for selected sites and the analogous transition for neutral sites (evidenced by strong differentiation and LD evolving for the latter). We emphasize that these predictions should be applied cautiously, because (i) a good deal of parameter space remains to be explored, and (ii) models are usually much cleaner than real systems.

To begin to attempt to connect empirical data and theory, we compared the general (qualitative) patterns from our simulation results to previously published population genomic data sampled from *Heliconius* species spanning various stages of evolutionary divergence along the speciation continuum [31]. A taxonomic pair’s position on this continuum may be characterized by the amount of genomic divergence and/or by the degree of reproductive isolation. Here, we focus on the former. In *Heliconius*, there is strong evidence of divergence with gene flow [31,32,33,34,35,36], despite multiple forms of reproductive isolation arising as both direct [37,38,39,40] and indirect [41,42,43,44,45] consequences of selection on mimetic Müllerian wing color patterns. We thus hypothesized that regions of the genome involved in maintaining barriers to gene flow would show the strongest evidence of coupling. Accordingly, we predicted that (1) evidence for coupling would be most pronounced for putatively selected loci, which we identified as outliers of the fixation index ($F_{ST}$) in the empirical data. However, given that gene flow might not have been continuous, we interpret these patterns cautiously. In addition, we predicted that (2) evidence of coupling between recently derived *Heliconius* species, differing only in aspects of color pattern (due to selection on only a single or small number of loci; [46]), would be restricted to chromosomes housing color patterning genes. We also predicted that (3) more extensive coupling across the genome would be observed as phylogenetic distance increased (reflecting reduced effective migration resulting from ongoing selection on color pattern and other forms of ecological divergence). In a purely allopatric scenario, neutral sites would readily diverge (between demes) along with the selected sites, due to the effects of hitchhiking and drift. However, even with small amounts of gene flow and recombination, neutral divergence would be delayed until after barrier loci had already become strongly coupled and diverged. Hence, we predicted that (4) the genomic background would show little divergence in most of our comparisons of taxa and regions of the genome. However, for taxa showing the greatest divergence (i.e., those farthest along the speciation continuum), the effects of coupling should be detectable even in the genomic background (i.e., the non-outlier sites).

## 2. Materials and Methods

### 2.1. Simulations

We performed forward-time, individual-based simulations with *bu2s* versions 3.6.1 and 3.7.1. The details of this software have been previously described [15,18]. Source code is freely available at *GitHub* [47]. Datasets used in this publication are archived at: http://bit.ly/2s6jeIf. The main extensions of the model not present in previous versions [18] are (i) the addition of neutral sites and (ii) the calculation of within-deme LD (previously, only between-deme LD was calculated). Since the rest of the workings of the *bu2s* model have been described in multiple previous publications [15,18,23], we give only a brief overview of *bu2s* here, focused on key elements and new features, and we refer the interested reader to those previous works for more details.

Table 1 summarizes key parameters, and their values, of the results. Parameters were chosen to include cases with s>m (per locus), in which divergence of individual loci is expected to be relatively smooth and linear in time, as well as cases with s<m, in which abrupt, nonlinear transitions in divergence and coupling are expected and individual mutations are often swamped by migration [18,23,48]. We used a total population size of 5000 because this allows for drift and enables simulations to run relatively quickly, but is also large enough to reveal central tendencies in evolutionary outcomes and population genetic statistics. Altogether, we show results from a total of 450 simulation runs, with 50 runs each of nine different parameter combinations (Table 1 and Table 2). Space limitations prevent us from presenting many additional parameter combinations. We thus emphasize that our results here are far from comprehensive, and a great deal of further exploration is warranted.

#### 2.1.1. Model Overview and Life Cycle

**Figure 1:**
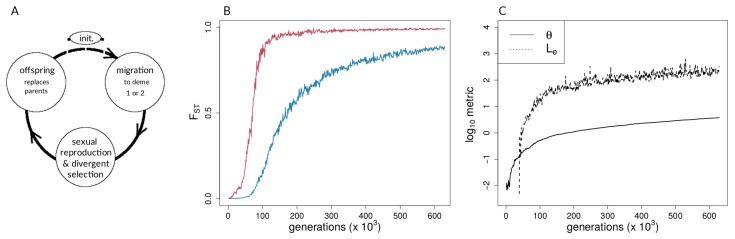
Forward-time simulations with (**A**) life-cycle diagram for the simulation model used, (**B**) population differentiation (fixation index, $F_{ST}$) over time for neutral (blue) and selected (red) sites, and (**C**) coupling measures for single simulation run. In (**B**,**C**), s=0.005 and m=0.01. The Methods section provides definitions of the coupling coefficient, θ, and the effective number of loci, $L_{e}$.

*bu2s* is a forward-time, mutation-based, stochastic model of divergence with gene flow, starting from a point with zero differentiation and zero segregating variation. Space is discrete, there are two demes, and individuals are diploid, hermaphroditic, and obligate outcrossers. Evolution results from the combination of mutation, selection, migration, recombination, and drift. In this setup, new mutations can either be neutral or subject to divergent selection. A life cycle diagram of the *bu2s* model is shown in Figure 1A.

#### 2.1.2. Migration

In each generation, each individual can migrate to the other deme with probability *m*.

#### 2.1.3. Selection, Fitness, and Reproduction

Following migration, reproduction occurs with the $N_{k}$ parents in deme *k* (*k* = 1 or 2) giving rise to $N_{k}$ offspring in that deme (i.e., soft selection). Selection occurs during reproduction: an individual’s relative fitness is linearly proportional to the probability that it contributes a gamete to the formation of each offspring. Fitness is calculated multiplicatively across selected loci, with each locus’ contribution to fitness given by Table 3. As the fitness scheme in Table 3 implies, fitness contributions of the alleles at a locus are additive (within the locus), and this is a “two-allele” model of barrier loci (sensu [5]). Though fitness is multiplicative between loci, there is no epistasis in the sense of incompatibilities.

In Table 3, $A_{i}$ is the ancestral allele at locus *i*, $B_{i}$ is the derived allele at locus *i* (originated by mutation during the simulation), and $s_{i}$ is the selection coefficient associated with locus *i*. It should be noted that a “locus” is synonymous with a “site” in this infinite sites model; any “locus” is a spot in the genome analogous to a single nucleotide polymorphism (SNP) in modern genomic sequencing data. The fitness of the *j*th individual, $W_{j}$, is calculated as

(1) $$W_{j}=\prod_{i=1}^{L}w_{ij}\left(g_{ij}\right)$$

where $w_{ij}\left(g_{ij}\right)$ is the contribution of locus *i* to individual *j*’s fitness, as a function of its genotype at that locus, $g_{ij}$ (Table 3), *L* is the total number of selected loci with segregating variants, and the product is over all such loci (i.e., ignoring neutral sites).

#### 2.1.4. Recombination

Recombination occurs during gamete formation (meiosis). Recombination locations are individually identically distributed along the length of the genome. In the results shown here, there were four chromosomes of equal length, with each chromosome having an expected number of 0.5 recombination events per meiosis (i.e., each was 50 centiMorgan (cM) long). The total number of recombination events thus follows a Poisson distribution with a mean equal to the length of the entire genome expressed in Morgans (=2 for results shown here).

#### 2.1.5. Mutation

A fixed number of mutations are introduced to the population in each generation (10/generation in results shown here). Each mutation is introduced in a randomly chosen offspring at a uniformly randomly chosen location in the genome. In results shown here, neutral and divergently selected mutations were introduced in a 10:1 ratio (i.e., a new mutation could be neutral with probability ∼0.909 or subject to divergent selection with probability ∼0.0909). Given that neutral mutations are more likely to be lost by drift, this ratio was chosen to allow a large number of both types of variants to build up standing variation. Additionally, it is realistic to expect more mutations to be neutral than beneficial. While the 10:1 ratio is likely to be biased in underrepresenting the true rate of neutral mutations for many taxa, a full-length simulation run would introduce well over 13.6 million neutral mutations, making it likely that even very rare events involving neutral mutations would occur. This, combined with the continuous genomic distribution of mutations, creates a huge variety of situations for indirect interactions between selected and neutral loci to occur and shape aggregate patterns.

Global positive selection and epistatic incompatibilities were not considered, in order to focus on the effects of genome-wide, divergent adaptation. Background selection was also ignored; however, a mutation may be disadvantageous in the population where it arises. Thus, this is a model in which reproductive isolation evolves by divergent adaptation only. Selection coefficients, $s_{i}$, are drawn from an exponential distribution with mean *s* (see [15,18] for discussion). All mutations arise de novo; there is no standing variation at the beginning of a simulation.

#### 2.1.6. Data Derived from Simulations and Metrics Computed from Simulation Data

Each simulation ran outputs standard population genetic metrics as time series, including global and deme-specific allele frequencies, $F_{ST}$, allele frequency spectra, samples of individual fitness values, and more (see [18] for additional metrics). $F_{ST}$ is calculated as $F_{ST}=\left(H_{T}-H_{S}\right)/H_{T}$, where $H_{T}$ is the total observed heterozygosity at a given locus at a given time step and $H_{S}$ is the expected heterozygosity based upon each deme’s observed heterozygosity [49]. One way that reproductive isolation can be measured in this model is by the expected effective backward migration rate [50], $m_{e}$, defined as the expected proportion of reproduction in a deme attributable to immigrants. This quantity shrinks as populations become increasingly isolated due to differential adaptation (i.e., as immigrants have lower and lower fitness relative to residents). For results shown here, simulations were run until 15 million mutations had been introduced (a maximum feasible runtime of about 168 h of CPU (central processing unit) time in some parameter combinations) or until effective migration dropped below an a priori threshold (whichever came first). We arbitrarily defined the latter threshold as $Nm_{e}<$ 0.0001, i.e., a less than 1 in 10,000 chance of having a single immigrant successfully reproduce. This means that, late in simulation runs, there are likely thousands of consecutive generations with zero effective migrants. We ran 450 simulations under different combinations of *s* and *m*, (Table 1), yielding 50 independent replicates of each specific parameter combination.

To connect our simulations to theoretical expectations about the fate of selected and neutral alleles derived from seminal work on hybrid zones, we calculated Barton’s coupling coefficient [4], defined here as $\theta \left(t\right)=\frac{\bar{s}\left(t\right)}{\bar{r}\left(t\right)}$. The dependence of θ on time is made explicit here to draw attention to the fact that, unlike analytic work, in the *bu2s* model, θ is not a fixed parameter but rather a dynamic variable that is a function of time. For notational simplicity, however, we refer to θ(t) henceforth as simply θ. θ changes in time for the following reasons. While *s* is indeed a fixed parameter in our simulations (Table 1), we note that it is the mean of a distribution from which mutation effect sizes, $s_{i}$, are drawn. The mutations that actually establish, especially early in a simulation, will be a non-random set of these because those with large values of $s_{i}$ will have a greater probability of establishment. Thus, the value of “$\bar{s}\left(t\right)$” used in our calculations of θ is the arithmetic mean of the segregating, selected alleles present at time *t* rather than the fixed value of the parameter *s* per se. Henceforth, we denote the parameter (fixed value) as *s*, and we denote the mean of the $s_{i}$ that are actually present at a given time as $\bar{s}$; likewise, $\bar{r}\left(t\right)$ is the arithmetic mean map distance between consecutive selected sites in the genome present at time *t*. $\bar{r}\left(t\right)$ varies for two reasons: (i) the number of segregating sites varies, and (ii) the locations of segregating sites vary (new mutations can arise anywhere in the genome). Additional explanations of these metrics and their calculation for the *bu2s* model can be found in the online supplementary material associated with Nosil et al. [51]. The coefficient θ is a quantitative description of the potential for coupling in the system. The actual amount of coupling at a given time can be measured by the effective number of loci, $L_{e}$ [4], which, in our discrete-space model, is the number of barrier loci that would have to be maximally coupled (i.e., in maximum linkage disequilibrium) to produce the observed between-deme difference in allele frequencies of barrier loci. Mathematically, following [4], $L_{e}$ is computed as

(2) $$L_{e}=\frac{s^{*}}{\bar{s}}\;$$

where $s^{*}$ is the value of the selection coefficient for a single locus that would cause the alleles of that locus to have the observed deme-specific frequencies at a migration-selection balance. In intuitive terms, $s^{*}$ captures the combined effect of direct and indirect selection on a locus. If all barrier loci are evolving independently (i.e., completely uncoupled; in linkage equilibrium), we expect alleles at each locus to exhibit single-locus migration-selection balance, which would result in $s^{*}∼\bar{s}$, giving $L_{e}∼1$. As loci become coupled, their alleles aid each other in reaching higher frequencies in their favored deme, causing departures from single-locus migration-selection balance such that $s^{*}>\bar{s}$ and associated increases in $L_{e}$ are above unity. $L_{e}$ will thus also indicate linkage disequilibrium among selected loci.

The calculation of $\bar{s}$ is described above. The calculation of $s^{*}$ is as follows. For the fitness scheme used in the *bu2s* model, and considering a single locus independently of all other loci, the equilibrium frequency of an allele under migration-selection balance in the deme in which it is favored is

(3) $$p_{i}=\frac{m\left(0.5+0.75s_{i}\right)-0.125s_{i}-0.125\sqrt{s_{i}^{2}-4ms_{i}^{2}+4m^{2}{\left(2+s_{i}\right)}^{2}}}{\left(-0.25+m\right)s_{i}}\;.$$

(Ref. [23], a *Mathematica* notebook with this solution and its derviation, can be downloaded from [52]). Let $\bar{p}$ be the mean observed frequency of barrier alleles in their favored deme. Substituting $\bar{p}$ for *p*, and $s^{*}$ for $s_{i}$ in Equation (3), we can then rearrange that equation to solve for $s^{*}$, yielding

(4) $$s^{*}=\frac{m(\bar{p}-0.5)}{\left(0.25-0.25*\bar{p}\right)\bar{p}+m\left(0.5+\bar{p}\left(\bar{p}-1.5\right)\right)}\;,$$

which provides the numerator in Equation (2) to calculate $L_{e}$.

For comparisons between simulation results and empirical data, we focused on statistics that are indicative of coupling and its effects, namely, linkage disequilibrium (LD). For the simulation results, LD was calculated as the correlation of allelic states (a value between 0 and 1; empirical LD methods described below). This was done for two different sets of loci: (i) the average pairwise value of LD between selected sites across the genome, and (ii) the average pairwise value of LD between neutral sites across the genome. In both cases, LD was calculated over the whole population, i.e., across demes (hereafter referred to as between-deme LD). We also calculated LD within each deme, pairwise between loci. The within-deme LD values were calculated from additional simulations conducted with an updated version of *bu2s*, 3.7.1. We used *R* [53] for the calculations of θ with the additional package *rhdf5* [54] for efficient parsing of simulation runs (see [55]).

To quantify times and conditions at which transitions occurred in simulations, we fitted generalized logistic models to results on AFD and between-deme LD. This choice of functional form (a logistic form) works well because AFD and LD are each bounded on the interval [0,1]. Below, we plot AFD as a function of θ, and the median of between-deme LD across runs as a function of time. For the former, we fit the data using

(5) $$y\left(x\right)=\frac{1}{\left(1+e^{-a(x-b)}\right)}$$

where *y* is AFD, and *x* is $\log_{10}\left(\theta \right)$. For LD as a function of time, we added one parameter to the logistic function, namely the asymptote (*z*), yielding

(6) $$y\left(x\right)=\frac{z}{\left(1+e^{-a(x-b)}\right)}$$

where *y* is the median between-deme LD across simulation runs and *x* is time. For both θ versus AFD and between-deme LD versus time, the coefficient *b* provides an estimate of the value of θ or the time at which the change in AFD or LD is most rapid (the inflection point of the logistic curve). The coefficient *a* is a shape parameter, with larger magnitudes of *a* indicating a steeper slope at the inflection point. Comparing values of *a* between selected and neutral sites gives a measure of how quickly each type of site undergoes a transition (once it has already begun). The value of *b* gives a measure of how long it takes transitions to begin, and comparing values of *b* provides an estimate of the difference or lag between transition points for the types of sites. Model fitting was performed with the *nls* function in *R* version 3.4.3 [53].

### 2.2. Empirical Data and Analyses

#### 2.2.1. Genotyping and Descriptive Population Genetic Statistics

**Figure 2:**
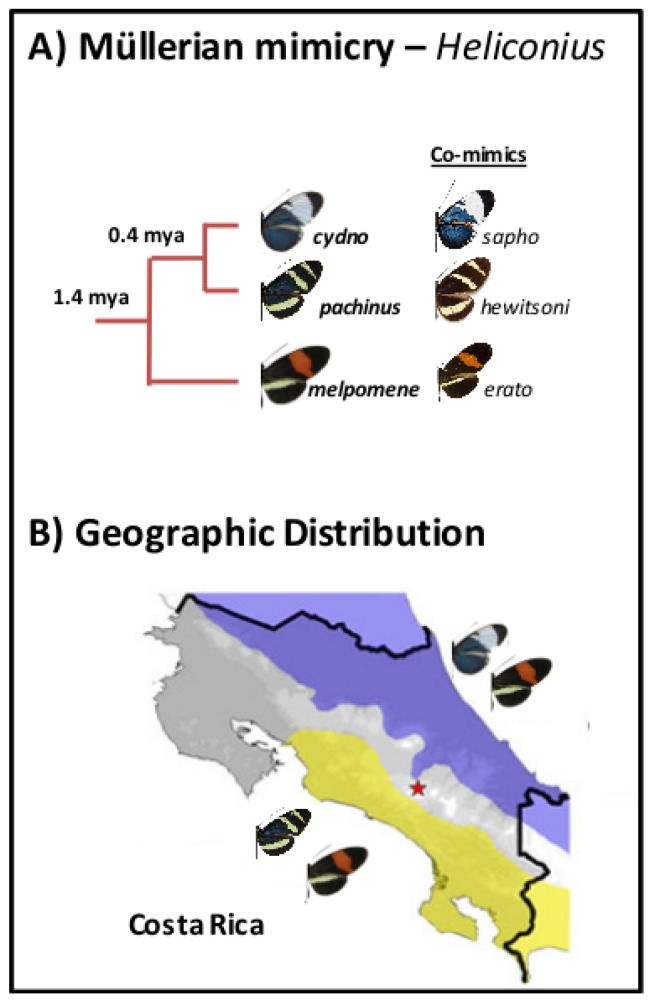
(**A**) Phylogenetic tree for *Heliconius* taxa and (**B**) geographic distribution in Costa Rica, with *Heliconius melpomene rosina* and *Heliconius cydno galanthus* occurring in sympatry on the Caribbean coastal drainage, and *Heliconius pachinus* and *H. melpomene rosina* co-occurring on the Pacific coastal drainage.

We used whole genome resequencing data, previously published by Kronforst et al. [31]. Individuals from three species were sampled in Costa Rica. *Heliconius cydno galanthus* & *Heliconius pachinus* (which are more closely related to each other than to *Heliconius melpomene rosina*) (Figure 2A) were sampled from the Caribbean and Pacific coastal drainages (Figure 2B), respectively (there is a contact zone between the two, which was not sampled there). *H. m. rosina* was sampled from overlapping sites with both *H. c. galanthus* and *H. pachinus*. Kronforst et al. [31] presented evidence of gene flow between the three species, and demonstrated signatures of (i) selection and adaptive introgression and (ii) elevated $F_{ST}$ on Z chromosomes. Additionally, they showed that known wing pattern loci are involved in initial divergence in these *Heliconius* species. We note that the set of samples does not include hybrids between any of the three species [31], so we do not necessarily capture the genetic variation across (continuous) clines. Previous work indicates very little population structure in the three focal taxa within Costa Rica [35], so we are confident that population stratification is not a concern.

Reads of *H. melpomene*, *H. c. galanthus*, and *H. pachinus* (*n* = 10 individuals/species) were aligned to the *H. melpomene melpomene* reference assembly version 2.5 [56] with *bwa mem* v.1.15.0 [57,58]. We used *GATK* [59] version 3.8 to call variants, with heterozygosity for prior likelihood calculation per locus of 0.001, and we ignored sequences with mapping quality <20. The minimum phred-scaled confidence threshold for variants to be called was set to 50. After genotype calling, we further filtered the data (see the respective *python* script name in [55] in parentheses) to only contain variants with a distance of >3 bp between neighboring variants *dropCloseVars.py*), and with depth of coverage ≤mean + 3*sd (*dropHighCovVars.py*). Further, we kept variants with a minimum absolute value of −8 in base quality rank sum tests, a minimum absolute value of the mapping quality rank sum test of −12.5, a minimum absolute value of the read position rank sum test of −8, a minimum ratio of variant confidence to non-reference read depth of 2, and finally a maximum phred-scaled *p*-value (using Fisher’s Exact Test to detect strand bias) of 60 (*vcfFilter.py*). Even with these steps of filtering, the number of sites retained was too large for exhaustive analysis. Hence, given the computational limitations and practical limitations of manuscript length, we focused on comparisons involving 5 chromosomes, which included two autosomes (2, 7) not implicated in color patterning, two autosomes containing extensively studied color patterning loci (10, 18) and the Z chromosome (21).

For each taxon and chromosome, we calculated nucleotide diversity (π) and Tajima’s D using *vcftools* [60] version 0.1.15. Additionally, for each of the three taxon pairs (*H. c. galanthus* & *H. m. rosina*; *H. pachinus* & *H. m. rosina*; *H. c. galanthus* & *H. pachinus*), we obtained estimates of absolute divergence ($d_{xy}$) and population differentiation ($F_{ST}$) [61] with *vcftools* for non-overlapping windows of a 10 kbp size per chromosome. We extracted genotype likelihoods (*vcf2gl.py*) for all included variable sites and further obtained the Bayesian posterior probability distribution for genotypes using EM estimates of population allele frequencies following [62] to empirically define Hardy–Weinberg priors. We then took the mean of the posterior for each locus (and individual) as a point estimate of the genotype, which is not constrained to be an integer, ranging from zero to two. From here on, we will refer to these as genotype estimates.

#### 2.2.2. Within-Species LD between *Heliconius* Loci

Based on genotype estimates, we calculated $F_{ST}$ (sensu [63]) and AFDs between taxa for each locus. In both cases, we calculated quantiles for each respective distribution, in order to determine sets of loci for the calculation of pairwise correlations between said loci. In the results section, we will focus on $F_{ST}$, since the procedure for $F_{ST}$ and AFDs is the same and the results for the AFD calculations were very similar to those seen for $F_{ST}$ (results based on AFDs can be found in the supplementary material). Loci were designated as outliers if $F_{ST}$ between two taxa lay above the 99th quantile for each chromosome. We expected outlier sites to be enriched for potential barrier loci, whereas non-outlier sites may represent putatively neutral sites but probably also contain weakly differentiated barrier alleles.

The resulting outliers are based on individual SNPs and not on windows containing multiple SNPs. We did this for two reasons: (i) we wanted to present a general approach that can be used in most systems, regardless of the quality of the genome assembly used to obtain SNPs, and (ii) using single SNPs for the calculation of within-species LD, without having to account for whether or not SNPs come in blocks of elevated differentiation, is practical. It will be interesting, however, to see whether the patterns of within-species LD for individual loci found here are similar for blocks of loci with increased differentiation.

**Figure 3:**
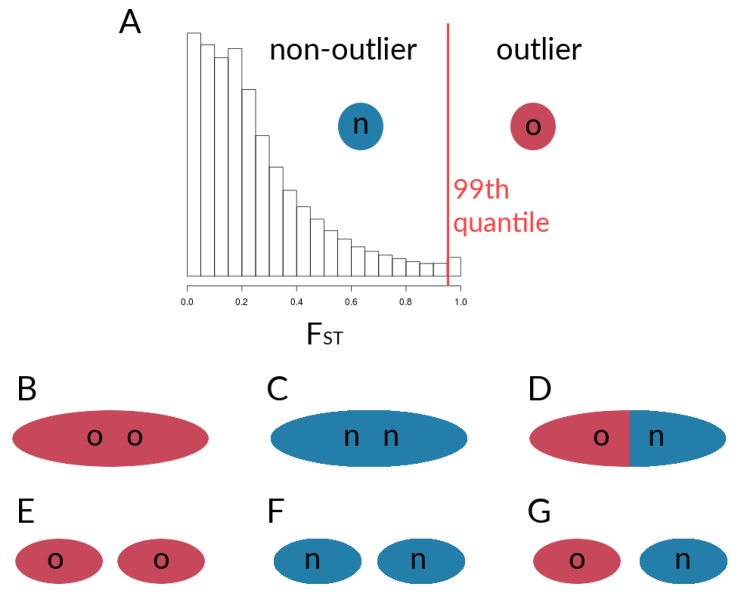
Schematic of comparisons for the calculation of correlation coefficients between sets of loci within species. (**A**) Designation of outlier loci based on $F_{ST}$, which was used for the calculation of $r^{2}$ for (**B**) outlier loci within the given chromosome, (**C**) non-outlier loci within the given chromosome, and (**D**) outlier loci with non-outlier loci within the given chromosome. Further, $r^{2}$ values werecalculated for sets of loci on the given chromosome with respective sets sampled from all other chromosomes, with (**E**) outlier loci vs. outliers from other chromosomes, (**F**) non-outlier loci vs. non-outliers from other chromosomes, and (**G**) outlier loci vs. non-outliers from other chromosomes.

After determining $F_{ST}$ outliers, we obtained estimates of within-species LD among different pairwise sets of loci (outliers and non-outliers), to find potential signatures of coupling between loci. Within each of the five chromosomes, as well as between chromosomes, we looked at simple measures of statistical non-independence. For outliers and non-outliers of both the single locus $F_{ST}$ values as well as the absolute AFD, we calculated Pearson’s correlation coefficients ($r^{2}$) of genotype estimates for six different groups of sites. It should be noted that the outlier designation is made from the comparison of two taxa; the values, however, then come from correlations calculated within each taxon. Here, we will use $F_{ST}$ outliers to illustrate the procedure (see also Figure 3). Of the six groups of sites considered here (Figure 3), there are three involved sites from the same chromosome, i.e., correlations that are (1) among outliers on the same chromosome (Figure 3B), (2) among non-outliers on the same chromosome (Figure 3C), and (3) between outliers and non-outliers on the same chromosome (Figure 3D). Additionally, we calculated pairwise $r^{2}$ values that are (4) between outliers on the given chromosome and outliers on all other chromosomes (Figure 3E), (5) between non-outliers on the given chromosome and non-outliers on other chromosomes (Figure 3F), and (6) between outliers on the given chromosome and non-outliers on the other chromosomes (Figure 3G). For the first two groups, we thus obtained pairwise correlation matrices. For the remainder of groups, however, we calculated $r^{2}$ for each of the target loci with genotype estimates of a sample of loci from the respective other set. For instance, for Group 3 (outlier vs. non-outlier within the given chromosome—Figure 3D), we randomly sampled genotype estimates of non-outlier sites. For each species and outlier site, we calculated correlation coefficients with said non-outlier sites, and sampled every second correlation value from the resulting correlation matrix, to obtain a total number of $r^{2}$ values equal to the number of elements in the upper triangle of the correlation matrices for Groups 1 & 2 (i.e., $\left(L_{o}^{2}-L_{o})/2\right)$, with $L_{o}$ = the number of outlier sites). For the correlations between SNPs on a given chromosome with loci on other chromosomes, we similarly sampled sites, although here, sites were sampled from all other chromosomes. Additionally, we limited the number of included SNPs per set to ≤5000. Subsampling was necessary, as the large number of variants would very quickly lead to vectors with sizes too large for downstream analyses. This sampling scheme still gave us more than 10 million correlation coefficients per respective set of loci. All of the coupling calculations as well as plotting were performed in *R* v.3.4.3 [53], with the additional package *ggplot2* [64]. Code to run these analyses can be found at [55].

## 3. Results

### 3.1. Investigating Coupling and Its Effects on Selected Versus Neutral Sites Using Simulations

**Figure 4:**
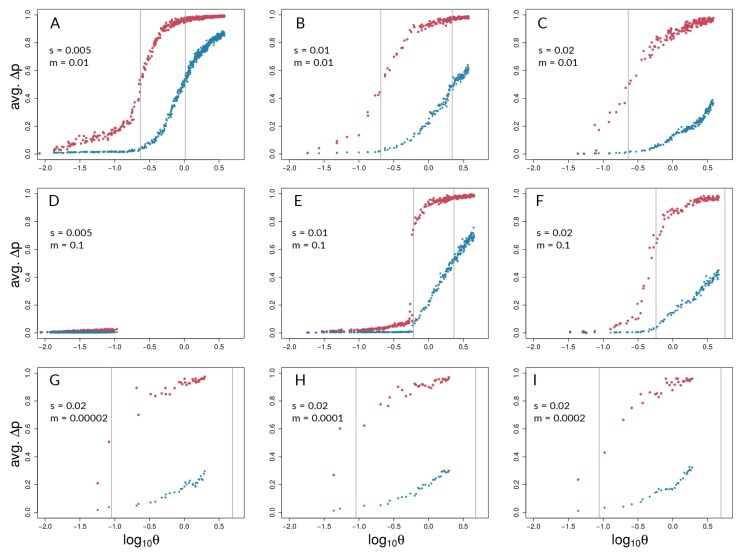
Barton’s coupling coefficient (θ) and average AFD between demes, for different combinations of *s* and *m* (values given in each panel). Each plot (A – I) shows data points for selected (red) and neutral (blue) sites from 50 independent simulation runs with equal parameters. Grey lines indicate the point of highest slope (i.e., inflection point) by nonlinear least squares model fitting across the 50 respective runs for selected and neutral sites (Table S2). It should be noted that grey lines could fall outside the range of respective data points if the predicted inflection point was not reached.

**Figure 5:**
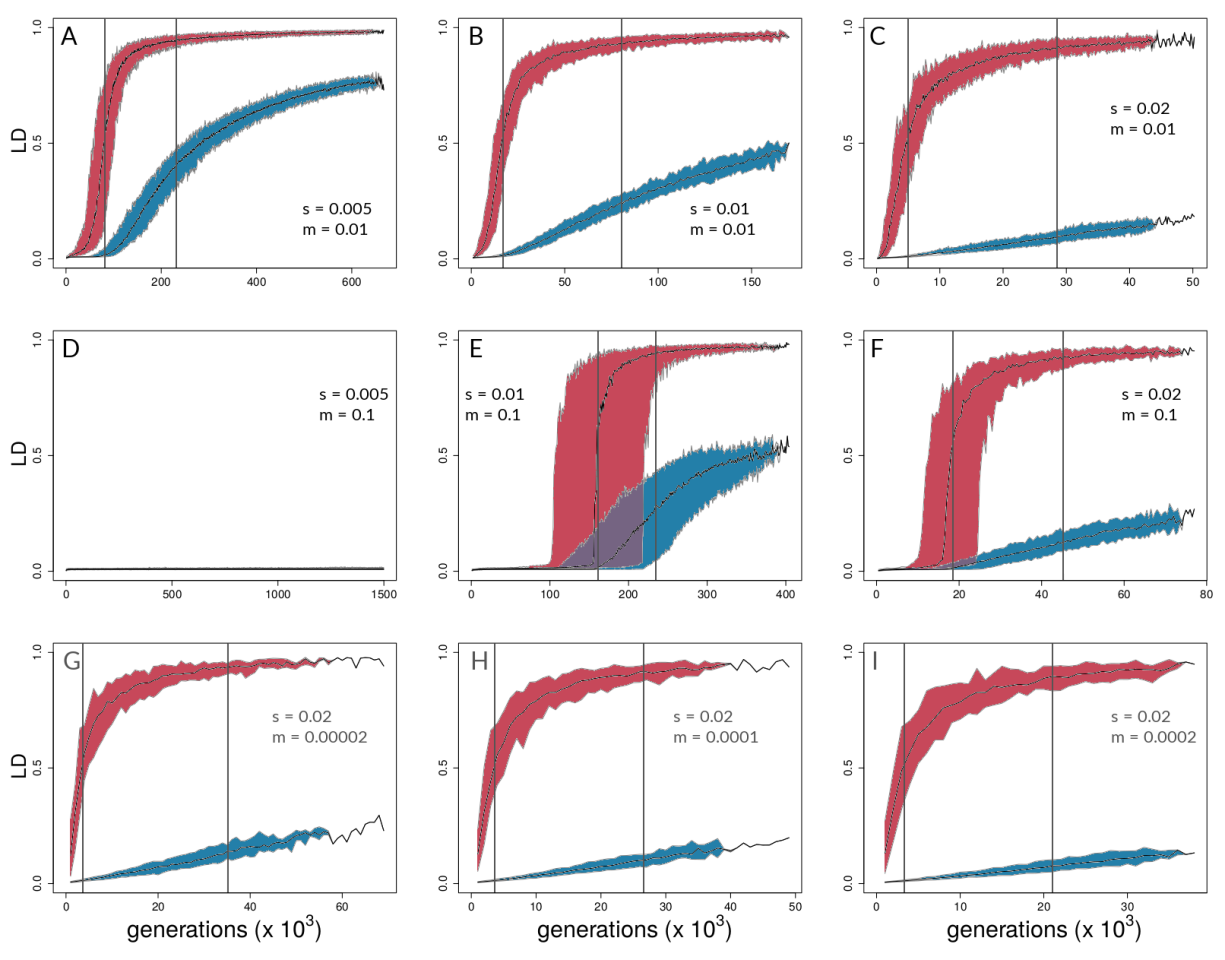
Time series of between-deme linkage disequilibrium (LD) for the same combinations of *s* and *m* as in Figure 4. Each plot (A – I) shows the median LD values across 50 independent simulation runs and 95% quantiles (for simulation medians) for selected (red) and neutral sites (blue). Grey lines indicate the point of highest slope by nonlinear least squares model fitting for medians across the 50 respective runs (Table S3 for details).

Of the 450 simulation runs with nine different parameter sets (with 50 runs each), only the 50 runs with *s* = 0.005 and *m* = 0.1 (i.e., runs with the highest ratio of m/s) did not reach the reproductive isolation threshold (which therefore ran for 1.5 million generations with 15 million mutations introduced); indeed, divergence never gained any traction in this parameter combination, and there were no hints of coupling (Figure 4D and Figure 5D). Aggregate summary statistics for all 450 simulations are given in the supplementary material (Table S1). It should be noted that, in Figure 4 and Figure 5, the first two rows of panels focus on scenarios with relatively high gene flow (Nm=50,500) and varying *s* (=0.005, 0.01, 0.02), whereas the bottom row of panels of each focuses on low gene flow scenarios (Nm=0.1,0.5,1) with the same value of *s* (=0.02).

A representative run with *s* = 0.005 and *m* = 0.01 is shown in Figure 1B,C and shows a lower migration compared with the above case. It can be seen that population differentiation ($F_{ST}$) between demes (Figure 1B) increased rapidly during the transition from single-locus to multi-locus divergence, where selected loci differentiated rapidly after ∼46,000 generations, and neutral sites showed strong differentiation after ∼75,000 generations (Figure 1B). By the time that reproductive isolation between demes reached our a priori threshold, in generation 629,000, selected sites had reached an average allele frequency difference between demes of 0.99, whereas neutral sites had reached an average allele frequency difference of 0.89. At this same point in time, $F_{ST}$ for neutral sites had not reached a maximum (or equilibrium) value. At the time when selected sites transition to being strongly coupled, estimators of coupling show a change in slope, and $L_{e}$ rises very quickly (Figure 1C). It should be noted that there are two dimensions of differences between selected and neutral sites seen in Figure 1B. First, the vertical distance between the points for selected and neutral sites at any point in time (i.e., at a given value on the *x*-axis) measures a gap in divergence at that time. We henceforth refer to these vertical distances as divergence “gaps.” Second, the horizontal distance between the selected and neutral points at a given level of divergence (i.e., at a given value on the *y*-axis) measures a lag in time. We refer henceforth to these horizontal distances as “lags” in divergence.

Aggregating runs and looking across combinations of selection and migration (Figure 4), selected sites can show strong coupling at values of the coupling coefficient, θ, for which neutral sites show negligible differentiation. This can be seen in all panels of Figure 4 except for Figure 4D,E by noting values of θ for which neutral sites have AFD very close to zero but selected sites have risen above zero. An exception is seen in Figure 4E, in which differentiation for selected and neutral sites takes off at about the same value of θ for each. This is the set of conditions (among those explored) in which differentiation was the most difficult but still possible (s=0.01<<m=0.1). Nonetheless, in Figure 4E, the time lag for neutral sites is still present to the maximal point of the slope differentiation, even though selected and neutral sites start gaining traction at about the same time.

Neutral sites began to show signs of differentiation for $\theta >10^{-0.5}\left(=\;∼0.32\right)$, regardless of the values of *s* and *m* (note where blue points begin to rise from zero in all panels of Figure 4 except Figure 4D). They reached their maximum rate of differentiation for values of θ≥1 (note the positions of the right-most vertical lines in Figure 4; there is no line shown for neutral sites in Panel C because it was off the scale; see Table S2 for exact values). Intuitively, selected sites showed signs of differentiation for lower values of θ compared with the neutral sites (note the red points above the blue points in Figure 4). Strong acceleration of differentiation indicates the occurrence of coupling among selected sites. The point of the maximum rate of change for selected sites as a function of θ depended strongly upon migration, *m*, and much less so upon *s*. When m=0.01, this point occurred for $\theta ∼10^{-0.65}$ (=0.22; see Table S2) regardless of *s* (Figure 4A–C). When *m* was increased, this point increased as well, to $\theta \;∼\;10^{-0.23}\left(=0.59\right)$, which again varied little with a two-fold change in *s* (Figure 4E,F). To summarize, there was a general association between how difficult it is for selected sites to differentiate and how similar the dynamics are for selected versus neutral sites, but there was no singular value of θ for which all scenarios showed transitions of either selected or neutral sites.

These differences between selected and neutral sites are borne out in time as well (Figure 5). Once there is sufficient potential for coupling, between-deme LD between selected sites increases rapidly in all parameter combinations that lead to divergence. LD across demes between neutral sites eventually shows increases in all these cases as well (Figure 5A–C,E–I), but the divergence gap between the neutral and selected sites is pronounced for long periods of time. The size of the gap (vertical distance between data points for selected and neutral sites in Figure 5) is related intuitively to the strength of selection: larger values of *s* lead to a greater gap in levels of divergence of selected versus neutral sites. This is seen by noting that the blue points reach lower levels as one moves from left to right across the panels within any one row of Figure 4 and Figure 5. However, it is important to note here that these gaps are not measured at the same point in time across all conditions. Rather, what we are highlighting here is relative to the time at which strong multi-locus coupling causes a transition to a highly differentiated state among selected sites (note the different time scales on panels of Figure 5). The lag between selected and neutral sites can be measured by the difference in inflection points of the fitted curves, i.e., where the rise in differentiation is predicted to be the steepest. This occurs for θ values that are consistently at least half an order of magnitude larger for neutral sites (compare *x*-axis distance between gray, vertical lines in Figure 4, and see Table S2). In time (Figure 5), this translates to 10s or 100s of thousands of generations, depending upon parameter values (see Table S3).

**Figure 6:**
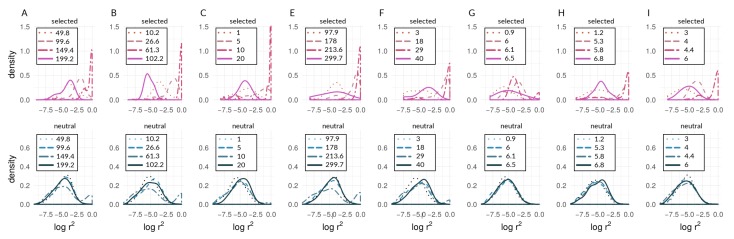
Density curves of within-deme LD as $\log(r^{2})$ for representative generations of *bu2s* runs (corresponding to Runs A,B,C,E,F,G,H, and I in Figure 4 and Figure 5), where (**A**) s=0.005, m=0.01, (**B**) s=0.01, m=0.01, (**C**) s=0.02, m=0.01, (**E**) s=0.01, m=0.1, (**F**) s=0.02, m=0.1, (**G**) s=0.02, m=0.00002, (**H**) s=0.02, m=0.0001, and (**I**) s=0.02, m=0.0002. Numbers in legends ($\times 10^{3}$) show the number of generations elapsed for respective lines and colors. Times vary from panel to panel because of the differing numbers of generations required for divergence with different parameters. In each case, the times chosen represent early, early-middle, late-middle, and late stages of divergence.

It should be noted that Figure 4D and Figure 5D underscore the existence of thresholds for transitions in divergence with gene flow: migration was so strong relative to selection (m=20s) in this scenario that sufficient standing variation in selected sites could never build up to raise θ high enough to cause coupling. Instead, selected and neutral variants were consistently eliminated in this scenario by the combination of migration and drift, resulting in an undifferentiated pseudo-equilibrium state of mutation-drift balance (true over all of the 50 replicates). Figure 6 shows pairwise LD of selected and neutral sites within one of the two demes in the forward-time simulations. Early during divergence, $\log(r^{2})$ are low (i.e., no strong correlations between pairs of loci-single-dotted lines in Figure 6), then we can see increasing values towards a maximum (dashed and two-dashed lines), and by the time they have diverged (so that immigrant alleles are too low in fitness to become established), pairs within demes exhibit low LD again (solid line in panels of Figure 6). Pairs of selected loci showed much stronger within-deme LD during this process than neutral ones, although it is visible in Figure 6 that, during the transition process, correlations between pairs of neutral loci show a second peak at higher values (tails of double-dashed blue curves in Figure 6A,B,E,F). This stands in contrast to patterns of between-deme LD, which over time shows an inflection before leading to an upper asymptote (see Figure 5). Within-deme LD values exhibit a different pattern (Figure 6), where values are decreasing, once gene flow between demes has effectively ceased.

### 3.2. Empirical Data and Analyses

#### 3.2.1. Genotyping and Descriptive Population Genetic Statistics

For all 21 chromosomes, we found a total of 12,739,517 variants after alignment to *Hmel 2.5*, genotyping, and further quality filtering. Across the five chosen chromosomes (2, 7, 10, 18, & 21), there are a total of 3,262,190 variants, with the number of variants and the number of scaffolds per chromosome given in Table S4.

Consistent with Kronforst et al. [31], we found that nucleotide diversity (π) was highest for *H. c. galanthus*, intermediate for *H. m. rosina*, and the lowest levels of polymorphism were found in *H. pachinus* (see Table S5 for values per chromosome and species). In all three taxa, π was consistently lowest on the Z chromosome (21). *H. c. galanthus* and *H. pachinus* showed lower levels of population differentiation from each other than from the more distant *H. m. rosina*, which can be seen from both levels of absolute divergence ($d_{xy}$) as well as $F_{ST}$ (see Table S6). Also consistent with Kronforst et al. [31], for both the comparisons involving the more distant *H. m. rosina*, values of $d_{xy}$ and $F_{ST}$ are considerably higher on the Z chromosome when compared to the autosomes, indicating higher species differentiation . For the remaining pair of *H. c. galanthus* with *H. pachinus*, however, we did not observe higher values of either $d_{xy}$ or $F_{ST}$ (see Table S6).

#### 3.2.2. Within-Species LD between *Heliconius* Loci

For the three pairs of taxa (i.e., *H. c. galanthus* – *H. m. rosina*, *H. pachinus* – *H. m. rosina*, and *H. c. galanthus* – *H. pachinus*) across the five chromosomes, we designated a total of 32,621 outliers above the 99th quantile of $F_{ST}$ (for AFDs, see SI). Quantiles of $F_{ST}$ across chromosomes showed average values of 0.97 (sd = 0.024) for the *c. galanthus* – *m. rosina* pair, 0.99 (sd = 0.013) for *pachinus* and *m. rosina*, and 0.429 (sd = 0.016) for *c. galanthus* and *pachinus*. Both species pairs involving *H. m. rosina* share many outlier sites (18.1% on the Z chromosome, and 27.9, 33, 27.2 and 26.9% for Chromosomes 2, 7, 10, and 18, respectively). On the other hand, outlier sites between *H. c. galanthus* and *H. pachinus* are predominantly private, i.e., do not occur in either of the pairs involving *H. m. rosina* (see also Figure S1).

**Figure 7:**
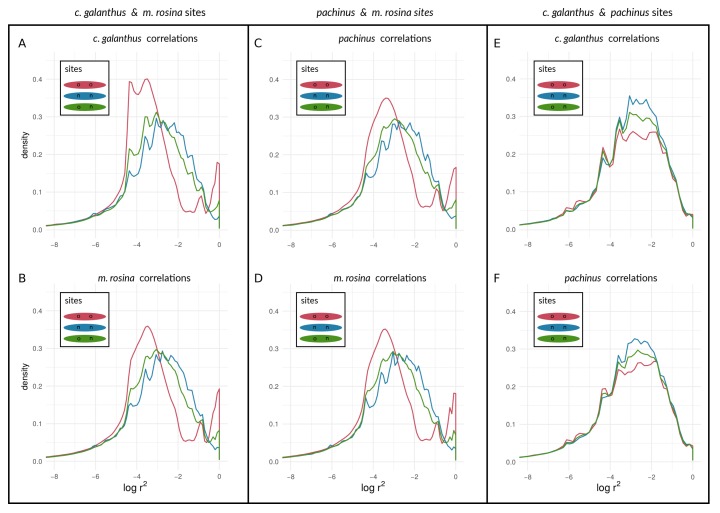
Density curves of within-species LD for loci at different types of sites on Chromosome 2, determined by $F_{ST}$ outliers. $Log\;r^{2}$ values are shown between outlier loci, between non-outliers, and between outliers and non-outliers for species pairs of *H. cydno galanthus* and *H. melpomene rosina* (**A**,**B**), of *H. pachinus* and *H. melpomene rosina* (**C**,**D**), and of *H. cydno galanthus* and *H. pachinus* (**E**,**F**).

**Figure 8:**
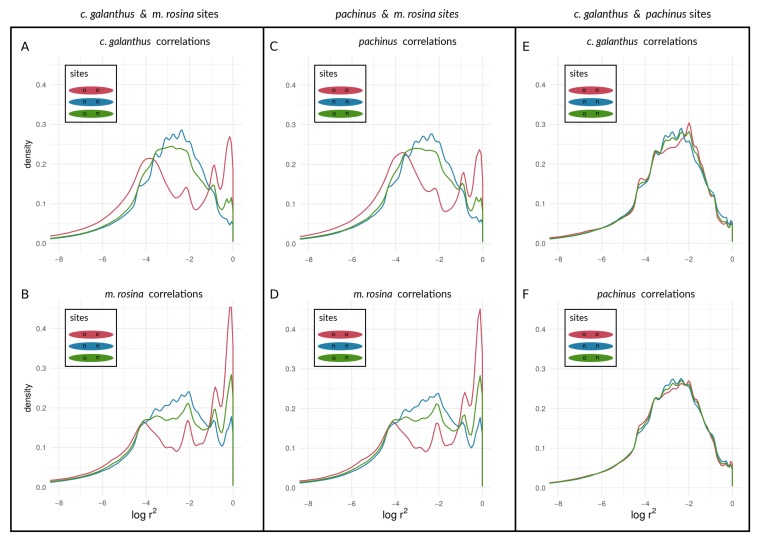
Density curves of within-species LD for loci at different types of sites on Chromosome 21, determined by $F_{ST}$ outliers. $Log\;r^{2}$ values are shown between outlier loci, between non-outliers, and between outliers and non-outliers for species pairs of *H. cydno galanthus* and *H. melpomene rosina* (**A**,**B**), of *H. pachinus* and *H. melpomene rosina* (**C**,**D**), and of *H. cydno galanthus* and *H. pachinus* (**E**,**F**).

We found a unimodal distribution of $\log(r^{2})$ between loci for most non-outlier sites. For the two taxon-pairs of (1) *H. c. galanthus* and *H. m. rosina*, as well as for (2) *H. pachinus* and *H. m. rosina*, we found bimodal distributions of $\log(r^{2})$ between outlier loci on autosomes (Figure 7A–D for Chromosome 2, and Figures S2–S4) and the Z chromosome (21) (Figure 8A–D), which deviate from the distributions of $\log(r^{2})$ between (1) non-outlier sites and (2) outliers and non-outliers. Furthermore, $\log(r^{2})$ between loci on the Z chromosome appear to have shifted to higher values, especially for correlations between outlier sites. The peak of neutral sites was smaller in the *H. m. rosina* outliers for both pairs involving *H. m. rosina* (Figure 7B,D), with a less pronounced peak at higher $\log(r^{2})$ values. In the third pair, *H. c. galanthus* with *H. pachinus*, we did not see a deviation of $\log(r^{2})$ between sites, neither for outliers nor for non-outliers in any of the autosomes (Figure 7E,F and Figure S2–S4) or the Z chromosome (Figure 8E,F).

Turning towards the coupling of respective sites with sites on other chromosomes, we found that both species pairs involving *H. m. rosina* showed evidence of coupling of outliers with outliers from other chromosomes—i.e., high values of $\log(r^{2})$—both for autosomes (Figure S5A–D for Chromosome 2; see also Figures S6–S8) as well as for the Z chromosome (Figure S9A–D). Similarly to the coupling within chromosomes, the *c. galanthus*–*pachinus* pair did not exhibit signs of coupling (Figures S5–S9E,F) with pairs of loci from other chromosomes. In plain terms, when a second, upper mode appears in a distribution of correlation coefficients, as seen in Cells A and B of Figure 7 and Figure 8 (see also Figures S2–S9), this is indicative of the occurrence of non-random associations between large numbers of sites. That is, the appearance of a second, upper mode indicates that a transition has occurred in which at least some portion of sites in the genome are statistically non-independent and thus coupled with each other. This is qualitatively different from seeing a (hypothetical) gradual shift in the mean and mode of a unimodal distribution. The system—and more specifically, coupled portions of the genome—has qualitatively shifted in its dynamics; a marked portion of the genome is now evolving as a coupled unit. This suggests that inversions and structural features are not the only reason for elevated LD between pairs of loci within species.

**Figure 9:**
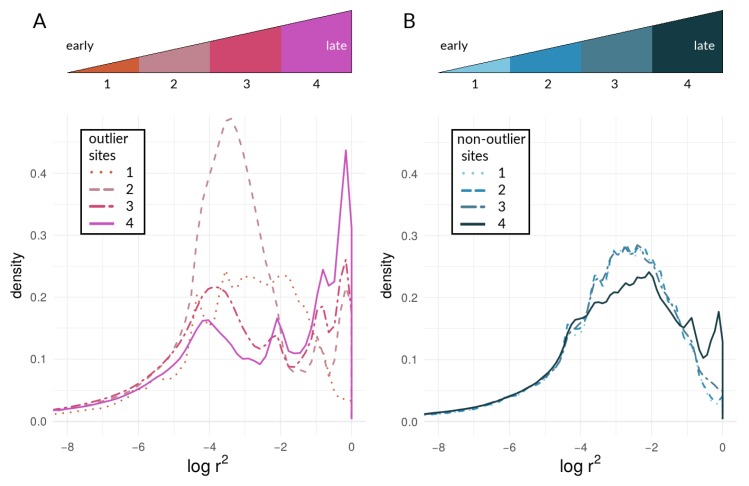
Density curves of within-species LD for (**A**) outliers and (**B**) non-outlier loci. Individual lines correspond to $\log(r^{2})$ values of (1) *pachinus* locus pairs for the comparison of *c. galanthus* and *pachinus* on Chromosome 2, (2) *m. rosina* locus pairs for the comparison of *pachinus* and *m. rosina* on Chromosome 2, (3) *m. rosina* locus pairs for the comparison of *c. galanthus* and *m. rosina* on Chromosome 21, and (4) *m. rosina* locus pairs for the comparison of *c. galanthus* and *m. rosina* on Chromosome 21. The upper panels illustrate the shift towards higher values of LD within species over time during the period of divergence with ongoing gene flow. Once gene flow has ceased (after 4), within-species LD is expected to decrease again. It should be noted that the stages depicted here do not fully correspond to the stages for the within-deme LD of our simulations (Figure 6). While a late stage has been reached in the simulations, and within-deme LD has already decreased, here, this has presumably not happened yet.

It should be noted that, with the sample sizes of numbers of outliers, there are thousands of sites near a given mode. Different types of sites (outliers vs. non-outliers), different chromosomes within a taxonomic comparison, and different taxonomic comparisons all show variation in the presence/absence and (if present) prominence of the upper mode of the distribution. We can hypothesize that this variation may indicate how different taxa and different portions of the genome are at different points along the speciation continuum. Under this hypothesis, these different points are proxies for changes in time, as Figure 9 attempts to illustrate. Moving from the curves in sequence from “1” to “4” in Figure 9A, we see increasing levels of coupling among outlier loci. Looking at the same comparisons for non-outlier sites in Figure 9B, we see the lag of non-outlier sites behind outlier sites, but that the non-outlier sites do eventually begin to show signs of being coupled as well, as evidenced by increased within-deme LD. Results based on AFD quantiles were very similar to $F_{ST}$ outliers (see Figures S10–S19 for all species pairs and respective chromosomes).

## 4. Discussion

Despite sustained and deep interest in the processes that lead to the evolution of diversity at multiple levels of biological organization, the study of speciation remains challenging because of the interacting mechanisms involved, the breadth and complexity of the potential demographic scenarios that encompass divergence, and the difficulty of integrating theoretical approaches with emergent empirical patterns observed in natural populations. The emphases on “speciation genes” or “islands of divergence”, in particular, have prevented more holistic and integrative considerations of genome-wide statistical patterns of divergence and gene flow throughout the speciation process in empirical studies of speciation (see also [23,65,66,67]). To address this limitation, we built upon previous theoretical results suggesting that LD among numerous weakly selected loci leads to rapid, genome-wide congealing during divergence by incorporating neutral sites into our model. In addition, we focused on classical signatures of multi-locus coupling, derived from hybrid zone theory (e.g., [4,9]), to investigate how divergent selection directly and indirectly affects the transition from independently evolving loci, which theory predicts should predominate early during speciation, to more extensive statistical linkage among loci at genome-wide scales. Finally, to investigate the qualitative agreement between the results of our theoretical simulations with patterns of divergence and LD in empirical data, we calculated measures of within-species LD for loci both within and between chromosomes for three species of hybridizing *Heliconius* butterflies representing both early and later stages of the speciation process. Studying the dynamics of neutral and selected sites in models is different from trying to distinguish between these classes of loci empirically. Thus, we need to be careful when interpreting such patterns in empirical data. Future work will have to show whether similar transitions occur in natural populations by combining forward-time simulations, coalescent simulations, and model-based parameter inference. In the meantime, we argue that it can be useful to examine the extent of coupling between loci, in addition to relative and absolute measures of divergence.

### 4.1. Simulations

We first investigated how patterns of divergence change over generations and over parameter conditions in our forward-time simulations of two demes under different levels of divergent selection and migration between demes (Figure 1, Figure 4, and Figure 5). In Felsenstein’s seminal paper [5], he argued that, in two-allele models, as presented here, the build-up of reproductive isolation should be constrained by migration because recombination is expected to break down associations among different selected loci. As seen here, however, if the repeated establishment of differentially adapted alleles is possible, a threshold can be reached at which strong divergence evolves and effective migration between demes is reduced, even at neutral sites [4,7,10,13,18,48] (Figure 4 and Figure 5). This transition is characterized by a shift from single loci acting independently to a joint effect of loci on the increasingly diverging genome. Such transitions in speciation occur when divergent selection and linkage disequilibrium reach a critical threshold, triggering genome-wide differentiation [18,23]. The impact of coupling on the barrier to gene flow at neutral loci has been documented both theoretically and empirically in the context of hybrid zones (e.g., [4,7,28,68,69]) but previous work has not thoroughly explored how the dynamics of transitions impact neutral sites temporally.

The results of our simulations suggest that, for the parameter space explored here, while both selected and neutral sites experience a similar build-up of genome-wide differentiation, selected loci, compared with neutral sites, experience this transition at lower values of θ (Figure 4) and earlier in time (Figure 5). Even when transitions for selected and neutral sites begin at nearly coincident points in time (e.g., Figure 5E) , the rates of increase in and magnitude of differentiation are quite different, as shown by the steepness and (for Figure 5) asymptotes of the fitted generalized logistic models (see Tables S2 and S3). These findings suggest that, for scenarios in which divergence happens with continuous gene flow, genome-wide statistical patterns may indeed offer substantial power to distinguish barrier from non-barrier loci, under a wide range of selection and migration conditions (e.g., across the panels of Figure 4 and Figure 5), at least once there is a transition to a state of multi-locus divergence (i.e., not in conditions like Figure 4D and Figure 5D). We emphasize that the simulations presented here focus on parameter cases with a specific (exponential) distribution of *s* values, and in which Nm lies between 0.1 and 500. Future explorations of a much broader set of parameter space are needed to determine if these preliminary findings from simulations are broadly generalizable. We also acknowledge that patterns in real systems are unlikely to be as clear-cut, regardless of parameters.

Although historically defined as the ratio of total selection and total recombination [4], the definition of coupling has recently been extended to include any process that leads to a coincidence of barrier effects and, hence, to stronger barriers to gene flow due to either the role of one-allele barriers or the build-up of LD among loci [3]. The use of a coupling statistic inspired by previous theory [4], θ, allowed us to encapsulate the potential for coupling in simulated systems. However, there was not a single approximate value of θ at which coupling of selected sites occurred. The sharp uptick in AFD, a dynamic change indicative of coupling, and the point of the maximum rate of change spanned a range of θ values (Figure 4). The uptick for differentiation at neutral sites was consistently at larger θ values, but it also varied . Comparing inflection points for neutral and selected sites as a function of θ, we observed the greatest difference in simulations with higher selection coefficients (*s*) and lower migration rates (*m*) (Figure 4C and Figure 5C; Table S2). That is, even with low rates of gene flow—indeed, even with a very low $m_{e}$ seen later in simulations—differentiation at neutral sites is very difficult to achieve and coupling among selected sites must be very strong to cut down gene flow at neutral sites. However, as *s* grows and/or *m* shrinks, it becomes easier and easier for selected sites to show divergence—and thus to rise in LD with each other as a statistical consequence—far in advance of the conditions at which neutral sites can diverge. It should be noted that these differences in threshold θ values do not translate into absolute differences in time; divergence for both types of sites is faster in absolute terms of time as *s* increases (*x*-axis scales in Figure 5).

Interestingly, the value of θ at which selected sites showed strong coupling and the most rapid differentiation depended strongly upon *m* but less so on *s*. This is seen by comparing the panels of Figure 4: in the upper row of panels, *m* is constant at 0.01, and although *s* is varied four-fold, the inflection point (coefficient *b* in model fits) stays at about the same value of θ (see Table S2). When *m* is increased but *s* is held constant (compare Figure 4B to Figure 4E, or Figure 4C to Figure 4F), the value of θ at the inflection point increases. One way to interpret this is that increases in *m* increase the effective amount of recombination between genotypes from different demes. As such, the potential for coupling, as encapsulated by θ, has to reach greater levels before coupling can actually occur.

We note that these predictions focus on divergence with gene flow. Though we did not study strict allopatric scenarios here, prior work [15,18] indicates that, for selected sites, when s>>m (as is true in allopatry when m=0), divergence from de novo mutations is relatively linear in time: differentiation between demes at selected sites will accumulate at a more-or-less constant rate dictated by the mutation rate, drift, and the strength of selection. For neutral sites, however, zero migration is likely to be very different than even small amounts of migration because—as results above indicate—even very low rates of effective gene flow and recombination are sufficient to prevent differentiation at neutral sites (i.e., neutral divergence did not show strong upticks until multi-locus barriers were very strong and the coupling coefficient reached high values). In a strict allopatry scenario, there would be no such impediment to neutral differentiation; thus, neutral sites would accumulate differentiation at a rate determined by mutation and drift, starting as soon as allopatry began. Furthermore, in a strict allopatry scenario, all de novo mutations would be private alleles; thus, between-deme LD would be at its maximum among all mutations that established, selected and neutral. This would produce very different distributions and time series of LD compared to our scenarios with gene flow.

Understanding additional quantitative dynamics of the differences between selected and neutral sites, including scenarios of strict allopatry as well as alternating periods of allopatry and gene flow, will require additional work. Further, we need to investigate the impact of the ratio of selected/neutral mutations and their genomic distributions in the simulated genomes on pairwise within-species LD. However, our results generally suggest that differences may exist in the emergence of significant LD among sites for selected and neutral loci at different stages of the speciation process and that heterogeneity in patterns of LD might reveal important details of where along the speciation continuum two hybridizing lineages currently reside.

### 4.2. Examining the Speciation Continuum in *Heliconius* by Using LD as a Proxy for Coupling

To empirically investigate speciation transitions related to coupling, we also assessed within-species LD among three *Heliconius* species pairs that are known to hybridize, spanning various stages of evolutionary divergence along the speciation continuum [31,35]. While LD is not required for coupling [3], it is expected when coupling occurs among loci that have “two-allele” effects (sensu [5]) in the presence of admixture. Specifically, we investigated patterns of within-species LD between different categories of loci identified as outliers or non-outliers based on population allele frequencies. Our implicit assumption is that outliers should be enriched for loci that act as barriers, such as those under divergent selection. However, we do not explicitly argue that non-outlier sites are neutral. Instead, we argue that the comparison of outlier sites with the genomic background (non-outliers which might have experienced varying direct or indirect selective pressures) represents a reasonable approach to investigate empirical patterns of divergence and coupling. We purposely chose to examine within-species LD among individual sites—rather than attempting to examine regions of differentiation (e.g., known color pattern loci in *Heliconius*)—to maximize the generalizability to other systems where knowledge about the specific targets of divergent natural selection leading to reproductive barriers may be unavailable.

Our approach was not meant to identify “speciation genes” per se. Rather, we examined the aggregate statistical properties—the statistical distributions of within-species LD values seen in Figure 7 and Figure 8—of chromosomes and the genome with respect to divergence. We argue that the categorical difference between a unimodal distribution (e.g., lines in Figure 7E) and a bimodal distribution (e.g., the red line in Figure 7A; see also Figure 6) could be a powerful indicator of populations and/or portions of the genome that are at different stages along the speciation continuum. Specifically, the appearance of an upper mode, close to the maximum possible value of within-species LD, is only plausibly explained by the coupling of barrier loci and its effects (though we emphasize that we are not attempting to infer the mechanism that produced this coupling in the empirical data). Notably, in the simulations, we observed an upper mode in the distribution of within-species LD for selected sites in only one scenario of parameter combinations (see Figure 6G, upper panel in red) but in several parameter combinations for neutral sites (see Figure 6A,B,E,F, lower panels in blue). In contrast, in *Heliconius*, we found an upper mode in all considered chromosomes for both selected and neutral sites in the *c. galanthus* and *m. rosina* comparisons as well as in the *pachinus* and *m. rosina* comparisons (see Figure 7, Figure 8, and Figures S2–S4). We are not able to say whether the upper modes seen for neutral sites in our simulations might be a consequence of the continuous distribution of sites in the model. This signals a gap in our current understanding, which needs to be addressed in the future.

Further, we found that signatures of within-species LD for outlier sites do coincide with prior findings of differentiation of both *H. c. galanthus* and *H. pachinus* with *H. m. rosina*. Moreover, within both of the two-species comparisons involving *H. m. rosina*, there is variation in within-species LD among different chromosomes, with the Z chromosome exhibiting the strongest signature of coupling (which is the most differentiated in both *c. galanthus* and *m. rosina* and the lowest nucleotide diversity (see Tables S5 and S6 and [31]). The third pair of taxa (*c. galanthus* and *pachinus*) does not exhibit signs of elevated within-species LD for the outliers. In other words, we found that the taxa that are more closely related to each other do not show LD within species, whereas taxa that have diverged further from each other, showed signatures of within-species LD, both within chromosomes as well as between chromosomes. Further, we found variation in within-species LD for different chromosomes, where the Z chromosome exhibits the strongest signal (coinciding with higher differentiation at the Z chromosome). The different patterns of within-species LD between different loci for the three pairwise species-comparisons could be seen as different stages along the speciation continuum and in the process of transitioning from uncoupled to coupled (Figure 9A), and this could be true for the genomic background as well (Figure 9B). Early on, allele frequencies are not very differentiated, and genotypes (within genomes) are not selected strongly as units. Thus, outliers (selected loci) can move and introgress as individual entities between taxa (with low within-species LD). However, when entering a tipping point and given enough differences between populations, indirect effects of selection between loci are starting to become more prevalent. Thus, the genome is slowly becoming the unit of selection, where migrant genotypes are starting to become eliminated as units. While this elimination of migrant genotypes does not happen immediately, the chance for individual alleles to “jump ship” decreases over time. Therefore, we see a second peak of pairs of loci, showing elevated LD within species (see Figure 7, Figure 8, and Figures S2–S4), even when they are physically unlinked between chromosomes (see Figures S5–S9). Finally, as taxa become extremely diverged, only a few lucky alleles will introgress. Alternatively, we could be observing historical introgression of a small number of alleles. As we move along the speciation continuum, we will see within-species LD decrease again, except for situations where there are structural features such as inversions and translocation polymorphisms that keep LD within taxa high for the loci they contain. In contrast to the within-species patterns, between-species LD will behave just like $F_{ST}$ (i.e., exhibit an inflection and eventually asymptote out). In both cases, however, neutral as opposed to selected sites will show qualitatively similar patterns, but they will differ in the timeframe and the magnitude of the pattern. This suggests an intriguing possibility. Namely, the characteristics of LD for selected and non-selected sites, within and between taxa, among chromosomes could be used to study the dynamics of how sites become coupled through time and act in concert to impede gene flow during the speciation process.

However, caution is warranted, when attempting to interpret the patterns of within-species LD during speciation. It is not clear whether different selection pressures or demographic histories could lead to similar patterns of LD as the ones seen here. It is also possible that sites in the genomic background—non-outliers that are less likely to be barriers—may also be under selection, and coupled, but simply haven’t reached high enough allele frequencies in the respective taxa, to be identified as outliers. Conversely, outliers may be enriched for neutral loci experiencing indirect selection, which could strongly affect patterns of linkage among sites. Another potential caveat to these results is that we estimated allele frequencies for each species using individual samples of each taxon rather than discrete populations. Thus, an unsampled population structure could potentially confound our allele frequency estimates. However, Kronforst and Gilbert [35] previously investigated these *Heliconius* species for evidence of population stratification, and found extremely low pairwise $F_{ST}$ between populations (*H. cydno*: 0.002–0.018; *H. melpomene*: 0.037–0.136; *H. pachinus*: 0–0.0016), corresponding with prior evidence of little population genetic structure based on allozyme data [70,71,72,73]. Another potential confounding factor to consider is the possibility that large structural variation (e.g., inversions; [20]) or other recombination modifiers [17,74,75,76,77] segregating between species might lead to higher estimates of LD within species. Feder et al. (2014) predicted that such modifiers would have to be large to be fixed in a population (i.e., on the order of megabases) [20]. However, recent work by Davey et al. (2017), using fine scale recombination maps, found no evidence of large-scale inversions between *H. melpomene* and *H. cydno*. Additionally, the occurrence of within-species LD between sites on different chromosomes (e.g., Figures S5–S9) would not be directly attributable to effects of structural variation (but is a good indicator of coupling across the entire genome). In the future, it will be interesting to see whether within-species LD can be used as a suitable summary statistic for understanding the role of two-allele coupling in speciation.

## 5. Conclusions

Our simulations focused on the de novo build-up of divergent adaptation, neutral differentiation, and coupling in the face of gene flow. We note, however, that the question and our results are also germane to cases of understanding the dynamics of speciation following secondary contact. Our findings pertain to the potential breakdown or retention and build-up of coupled complexes of selected and neutral genes following contact of diverged populations as well. Future theory will more explicitly explore the effects of varying periods of allopatry, ratios of selected/neutral mutations, and the genomic distribution of mutations. Even so, making explicit quantitative comparisons between simulation results and empirical data is difficult, as we do not have temporal data from *Heliconius* species spanning large numbers of generations. Future work will attempt to surmount this with a combination of forward-time simulations, coalescent simulations, and model-based parameter inference. Additionally, it would be particularly useful to broaden the scope to a wider range of *Heliconius* taxa, as well as to other cases of adaptive radiations for comparative purposes. With data from multiple independent radiations, we could test the generality and scale(s) of repeatability of patterns of divergence.

Coupling between loci represents an informative component of the study of the genetics of speciation [3]. We need to examine a breadth of additional systems to gain insight into the potential generality of coupling measures to reveal speciation in action. Additionally—recognizing that our simulations focused on only divergent adaptation and neutral processes here—it is crucially important that theory continues to broaden to include the interactions of a variety of types of selection (background, balancing, positive, epistatic, etc.) and their interactions with realistic demographic processes. Doing so will enable refinement of the predictions we can make about the signatures of the multitude of processes shaping genome-wide variation in natural populations.

## Figures and Tables

**Table 1:** Parameter values used in simulations. See Table 2 for the combinations of *s* and *m* values used.

Parameter	Notation (If Applicable)	Value(s) Used (and Units if Applicable)
Mean selection coefficient for divergently selected mutations (mean of exponential distribution from which new mutations’ coefficients were drawn)	*s*	0.005, 0.01, 0.02
Migration rate	*m*	0.00002, 0.0001, 0.0002, 0.01, 0.1 (probability per individual per generation)
Total population size	*N*	5000 individuals
Mutations per generation (population mutation rate)		10 per generation
Number of chromosomes in a genome (haploid number)	*c*	4
Recombination length of each individual chromosome	*l*	50 centiMorgan (cM)
Ratio of neutral:selected mutations		10:1

**Table 2:** The nine parameter combinations presented, and the corresponding figure panels, as denoted by letters, in Figures 4 and 5. Fifty replicates of each noted combination were used. “-” means that results were not generated for the given combination.

	*s*	0.005	0.01	0.02
*m*				
0.01		**A**	**B**	**C**
0.1		**D**	**E**	**F**
0.00002		**G**	-	-
0.0001		**H**	-	-
0.0002		**I**	-	-

**Table 3:** Fitness scheme for loci under selection.

Genotype at Locus *i*	Fitness Contribution of Locus in Deme 1	Fitness Contribution of Locus in Deme 2
$A_{i}A_{i}$	$1+s_{i}$	1
$A_{i}B_{i}$	$1+0.5s_{i}$	$1+0.5s_{i}$
$B_{i}B_{i}$	1	$1+s_{i}$

## Supplementary Materials

The following are available online at http://www.mdpi.com/2073-4425/9/6/274/s1: Table S1: Summary statistics for the 450 included simulation runs; Table S2: Summary for nonlinear least squares fit of Barton’s coupling coefficient (θ) and average allele frequency differences between demes for selected and neutral loci; Table S3: Summary for nonlinear least squares fit of average LD for selected and neutral loci; Table S4: Numbers of scffolds and variants for each invluded *Heliconius* chromosome; Table S5: Population genetic statistics for *Heliconius* species per chromosome; Table S6: Population genetic statistics for *Heliconius* species pairs per chromosome; Figure S1: Venn diagrams to illustrate the overlap for outliers of $F_{ST}$ and AFD; Figure S2: Density curves of within-species LD for loci at different types of sites on Chromosome 7, determined by $F_{ST}$ outliers; Figure S3: Density curves of within-species LD for loci at different types of sites on Chromosome 10, determined by $F_{ST}$ outliers; 5 Figure S4: Density curves of within-species LD for loci at different types of sites on Chromosome 18, determined by $F_{ST}$ outliers; Figure S5: Density curves of within-species LD for loci at different types of sites on Chromosome 2 and respective sites on all other chromosomes, determined by $F_{ST}$ outliers; Figure S6: Density curves of within-species LD for loci at different types of sites on Chromosome 7 and respective sites on all other chromosomes, determined by $F_{ST}$ outliers; Figure S7: Density curves of within-species LD for loci at different types of sites on Chromosome 10 and respective sites on all other chromosomes, determined by $F_{ST}$ outliers; Figure S8: Density curves of within-species LD for loci at different types of sites on Chromosome 18 and respective sites on all other chromosomes, determined by $F_{ST}$ outliers; Figure S9: Density curves of within-species LD for loci at different types of sites on Chromosome 21 and respective sites on all other chromosomes, determined by $F_{ST}$ outliers; Figure S10: Density curves of within-species LD for loci at different types of sites on Chromosome 2, determined by outliers of AFDs; Figure S11: Density curves of within-species LD for loci at different types of sites on Chromosome 7, determined by outliers of AFDs; Figure S12: Density curves of within-species LD for loci at different types of sites on Chromosome 10, determined by outliers of AFDs; Figure S13: Density curves of within-species LD for loci at different types of sites on Chromosome 18, determined by outliers of AFDs; Figure S14: Density curves of within-species LD for loci at different types of sites on Chromosome 21, determined by outliers of AFDs; Figure S15: Density curves of within-species LD for loci at different types of sites on Chromosome 2 and respective sites on all other chromosomes, determined by outliers of AFDs; Figure S16: Density curves of within-species LD for loci at different types of sites on Chromosome 7 and respective sites on all other chromosomes, determined by outliers of AFDs; Figure S17: Density curves of within-species LD for loci at different types of sites on Chromosome 10 and respective sites on all other chromosomes, determined by outliers of AFDs; Figure S18: Density curves of within-species LD for loci at different types of sites on Chromosome 18 and respective sites on all other chromosomes, determined by outliers of AFDs; Figure S19: Density curves of within-species LD for loci at different types of sites on Chromosome 21 and respective sites on all other chromosomes, determined by outliers of AFDs.
