# Supplementary material for: Transitions from Single- to Multi-Locus Processes during Speciation with Gene Flow
Source: Genes (Basel). 2018 May 24;9(6):274. doi: 10.3390/genes9060274 (PMC6027428; doi:10.3390/genes9060274)
Supplement: Supplementary file 1 [file genes-09-00274-s001.pdf]

Supplementary Materials

Transitions from single- to multi-locus processes during speciation

Schilling MP, Mullen SP, Kronforst M, Safran RJ, Nosil P, Feder JL, Gompert Z, and Flaxman SM

2018

Table S1: Summary statistics for the 450 included simulation runs.

set	A	B	C	D	E	F	G	H	I
s	0.005	0.01	0.02	0.005	0.01	0.02	0.02	0.02	0.02
m	0.01	0.01	0.01	0.1	0.1	0.1	0.00002	0.0001	0.0002
nRuns	50	50	50	50	50	50	50	50	50
mean number of variable loci	2386.02	1077.2	554.48	197.02	1202.46	626.3	555.82	490.68	470.9
sd number of variable loci	56.67	36.94	23.93	13.59	32.47	22.02	89.0	41.54	24.92
mean total generations elapsed	619540	155160	39979.68	1500000	333960	63230	38560	31940	31200
sd total generations elapsed	24938.46	8524.54	2970.84	0	32459.68	5090.22	7775.31	3893.53	2555.51
RI reached	1	1	1	0	1	1	1	1	1
mean total mutations introd.	6195409	1551609	399805.8	15000000	3339609	632309	385609	319409	312009
sd total mutations introd.	249384.6	85245.38	29708.39	0	324596.8	50902.17	77753.14	38935.32	25555.06

Table S2: Summary for nonlinear least squares fit of Barton's coupling coefficient (θ) and average allele frequency differences between demes for selected and neutral loci.

run set	A	B	C	D	E	F	G	H	I
s	0.005	0.01	0.02	0.005	0.01	0.02	0.02	0.02	0.02
m	0.01	0.01	0.01	0.1	0.1	0.1	0.00002	0.0001	0.0002
a selected	5.6098	3.9289	3.1885	1.5041	21.4833	9.2014	2.445	2.4584	2.4417
b selected	-0.6312	-0.6862	-0.6405	1.5251	-0.2158	-0.2409	-1.048	-1.0429	-1.0585
a neutral	3.8938	2.8471	2.3875	0.06	3.8226	2.6796	2.2129	2.1761	2.1855
b neutral	0.0141	0.3384	0.852	86.258	0.3645	0.7493	0.6911	0.6785	0.6930
p value	$< 2e^{-16}$	$< 2e^{-16}$	$< 2e^{-16}$	$< 2e^{-16}$	$< 2e^{-16}$	$< 2e^{-16}$	$< 2e^{-16}$	$< 2e^{-16}$	$< 2e^{-16}$
p value	$< 2e^{-16}$	$< 2e^{-16}$	$< 2e^{-16}$	$< 2e^{-16}$	$< 2e^{-16}$	$< 2e^{-16}$	$< 2e^{-16}$	$< 2e^{-16}$	$< 2e^{-16}$
s res. std. err.	0.0243	0.0231	0.0316	0.003	0.0557	0.055	0.0325	0.0344	0.0347
n res. std. err.	0.0302	0.0294	0.026	0.00059	0.0355	0.031	0.0288	0.0252	0.0259
df	30975	7756	11620	74948	16696	6321	1926	1595	1558

Table S3: Summary for nonlinear least squares fit of average LD for selected and neutral loci. Run D did not yield intelligible results and was excluded here.

run set	A	B	C	D	E	F	G	H	I
s	0.05	0.01	0.02	0.005	0.01	0.02	0.02	0.02	0.02
m	0.01	0.01	0.01	0.1	0.1	0.1	0.00002	0.0001	0.0002
z selected	0.962	0.935	0.905	–	0.948	0.918	0.9368	0.9162	0.9011
a selected	0.0614	0.1835	0.076	–	0.213	0.273	0.303	0.3455	0.3714
b selected	81.12	16.927	28.774	–	161.3	37.05	3.6779	3.6146	3.3015
z neutral	0.729	0.463	0.1892	–	0.508	0.245	0.2635	0.2019	0.1496
a neutral	0.0147	0.0361	0.0167	–	0.0314	0.0406	0.0724	0.0949	0.12
b neutral	231.6	80.5	166	–	234.6	90.06	35.1148	26.5898	21.025
p value	$< 2e^{-16}$	$< 2e^{-16}$	$< 2e^{-16}$	–	$< 2e^{-16}$	$< 2e^{-16}$	$< 2e^{-16}$	$< 2e^{-16}$	$< 2e^{-16}$
p value	$< 2e^{-16}$	$< 2e^{-16}$	$< 2e^{-16}$	–	$< 2e^{-16}$	$< 2e^{-16}$	$< 2e^{-16}$	$< 2e^{-16}$	$< 2e^{-16}$
s res. std. err.	0.0208	0.03326	0.0428	–	0.035	0.039	0.0392	0.0366	0.0417
n res. std. err.	0.0312	0.016	0.0063	–	0.015	0.0095	0.0146	0.0066	0.0042
df	664	167	289	–	401	151	66	46	35

Table S4: Numbers of scffolds and variants for each invluded *Heliconius* chromosome.

Chromosome	no. scaffolds	no. variants
2	1	449,499
7	1	667,749
10	1	850,334
18	3	771,594
21 (Z)	1	523,014

Table S5: Population genetic statistics for *Heliconius* species per chromosome.

species	chromosome	π (sd)	Tajimas D (sd)
c. galanthus	2	0.24 (0.06)	0.66 (0.60)
c. galanthus	7	0.23 (0.06)	0.59 (0.63)
c. galanthus	10	0.23 (0.06)	0.63 (0.62)
c. galanthus	18	0.23 (0.06)	0.63 (0.61)
c. galanthus	21	0.21 (0.06)	0.70 (0.66)
m. rosina	2	0.22 (0.07)	0.87 (0.76)
m. rosina	7	0.24 (0.07)	0.73 (0.88)
m. rosina	10	0.23 (0.08)	0.84 (0.88)
m. rosina	18	0.23 (0.08)	0.86 (0.85)
m. rosina	21	0.13 (0.07)	0.89 (0.92)
pachinus	2	0.22 (0.06)	0.83 (0.67)
pachinus	7	0.21 (0.07)	0.72 (0.75)
pachinus	10	0.20 (0.07)	0.73 (0.77)
pachinus	18	0.20 (0.07)	0.74 (0.75)
pachinus	21	0.18 (0.07)	0.81 (0.77)

Table S6: Population genetic statistics for *Heliconius* species pairs per chromosome

species pair	chromosome	F_{ST} (sd)	dxy (sd)	no. outliers (AFDs & F_{ST}, resp.)	99% quant. AFDs	99% quant. F_{ST}
c. galanthus - m. rosina	2	0.23 (0.25)	0.37 (0.09)	4495	0.9484	0.9513
c. galanthus - m. rosina	7	0.22 (0.25)	0.37 (0.10)	6677	0.9274	0.9499
c. galanthus - m. rosina	10	0.25 (0.26)	0.38 (0.09)	8503	0.9495	0.95
c. galanthus - m. rosina	18	0.25 (0.27)	0.38 (0.10)	7716	0.9967	0.9912
c. galanthus - m. rosina	21	0.39 (0.33)	0.46 (0.11)	5230	0.9963	0.9963
pachinus - m. rosina	2	0.27 (0.27)	0.38 (0.09)	4495	0.9848	0.9907
pachinus - m. rosina	7	0.27 (0.27)	0.39 (0.10)	6677	0.9573	0.9677
pachinus - m. rosina	10	0.30 (0.28)	0.40 (0.10)	8503	0.9912	0.9933
pachinus - m. rosina	18	0.29 (0.28)	0.40 (0.11)	7716	0.9967	0.9981
pachinus - m. rosina	21	0.44 (0.35)	0.48 (0.12)	5230	0.9963	0.998
c. galanthus - pachinus	2	0.09 (0.13)	0.26 (0.06)	4495	0.4152	0.4399
c. galanthus - pachinus	7	0.08 (0.13)	0.25 (0.06)	6677	0.3999	0.4112
c. galanthus - pachinus	10	0.09 (0.14)	0.24 (0.06)	8503	0.4233	0.4188
c. galanthus - pachinus	18	0.09 (0.13)	0.24 (0.06)	7716	0.4019	0.422
c. galanthus - pachinus	21	0.10 (0.15)	0.23 (0.06)	5230	0.4544	0.4495

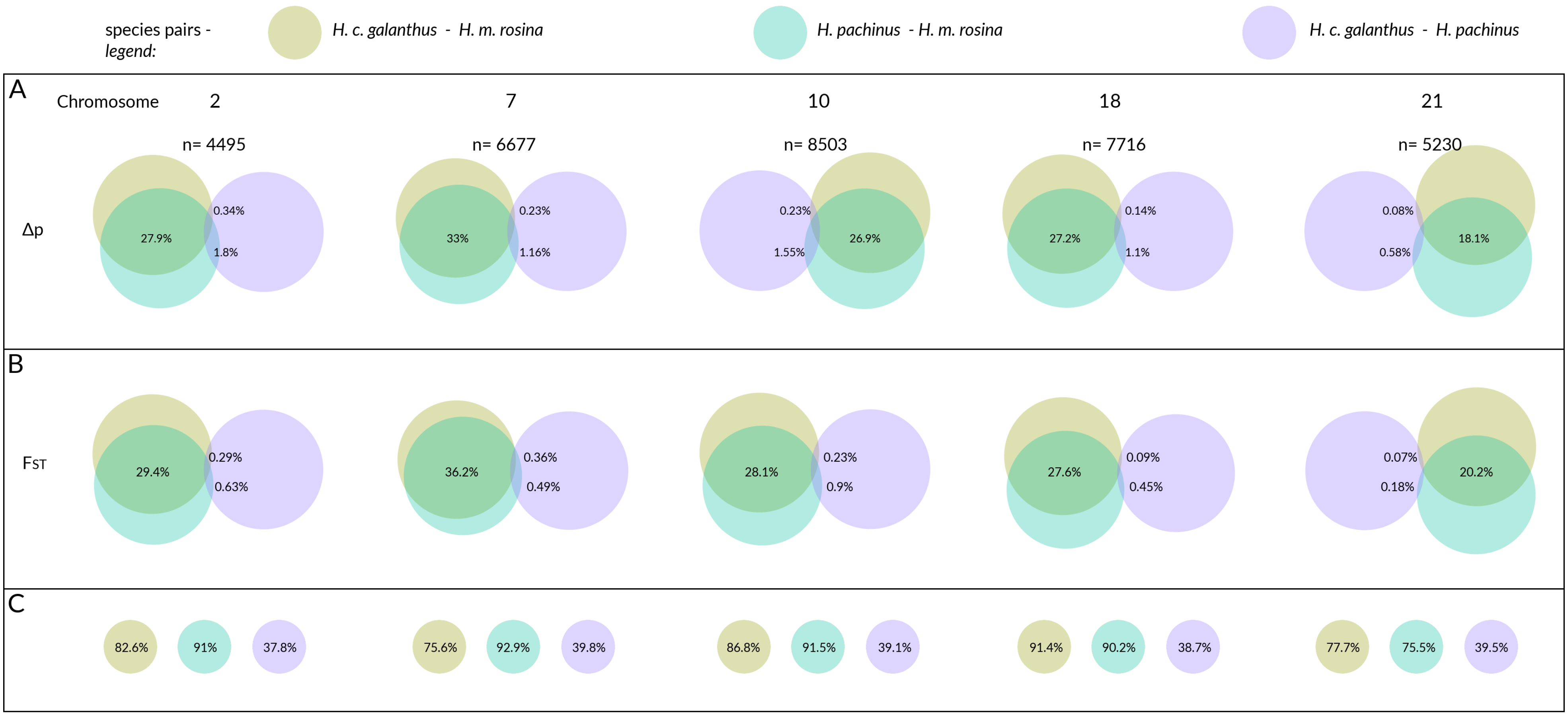

Figure S1: Venn diagrams to illustrate the overlap of outliers for A) AFDs and B) F_{ST} and all species pairs and chromosomes. Percentage of overlap is shown for respective pairs of taxa. C) Percentage of overlap between AFD and F_{ST} species pairs and chromosomes. This is not a Venn diagram, but simply shows the percentage between two sets in the circle.

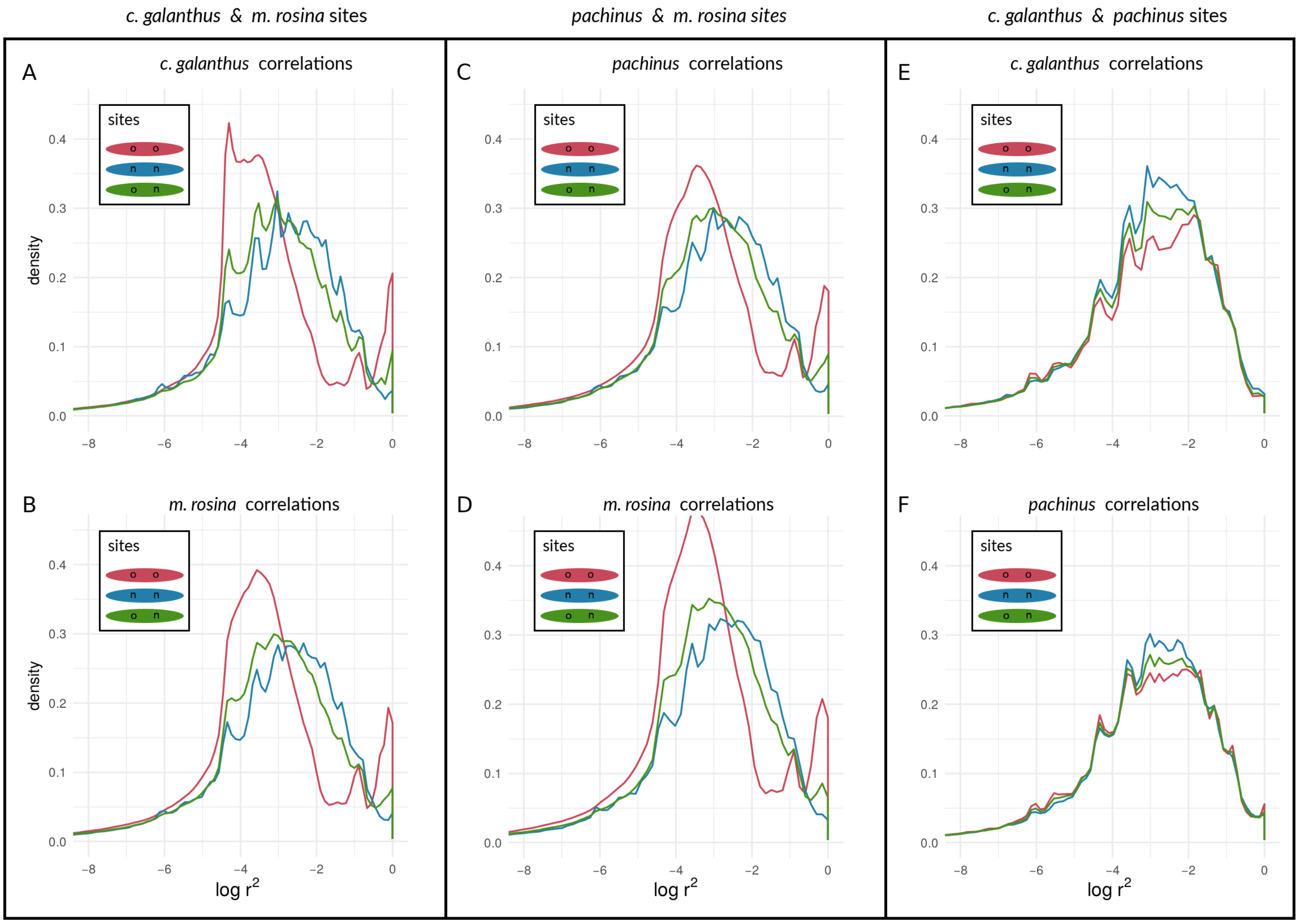

Figure S2: Density curves of within-species LD for loci at different types of sites **on chromosome 7**, determined by F_{ST} outliers. *Log* r^2 values are shown between outliers, between non-outliers and between outliers and non-outliers for species pairs of *H. cydno galanthus* and *H. melpomene rosina* (A & B), *H. pachinus* and *H. melpomene rosina* (C & D), and *H. cydno galanthus* and *H. pachinus* (E & F).

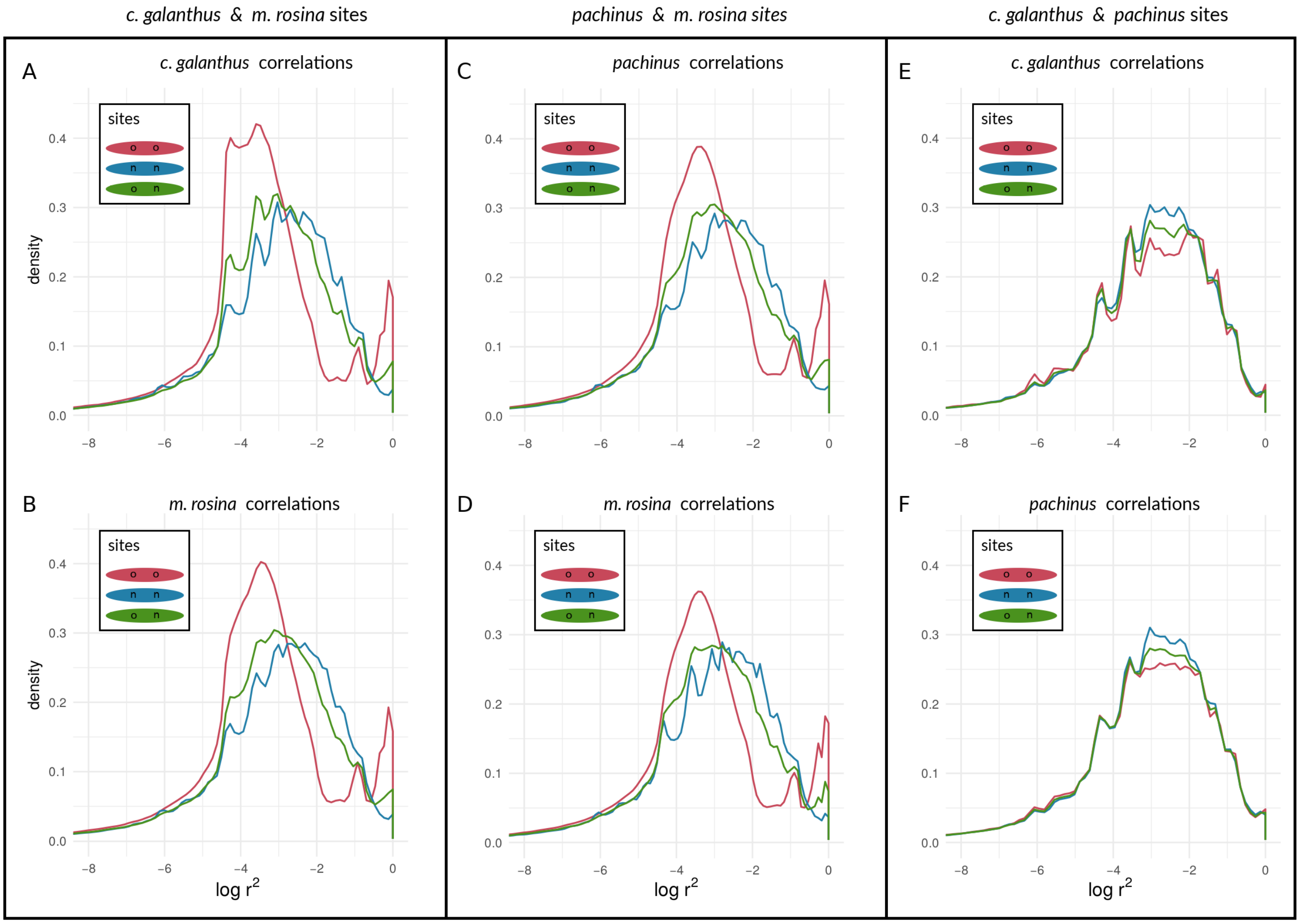

Figure S3: Density curves of within-species LD for loci at different types of sites **on chromosome 10**, determined by F_{ST} outliers. *Log* r^2 values are shown between outliers, between non-outliers and between outliers and non-outliers for species pairs of *H. cydno galanthus* and *H. melpomene rosina* (A & B), *H. pachinus* and *H. melpomene rosina* (C & D), and *H. cydno galanthus* and *H. pachinus* (E & F).

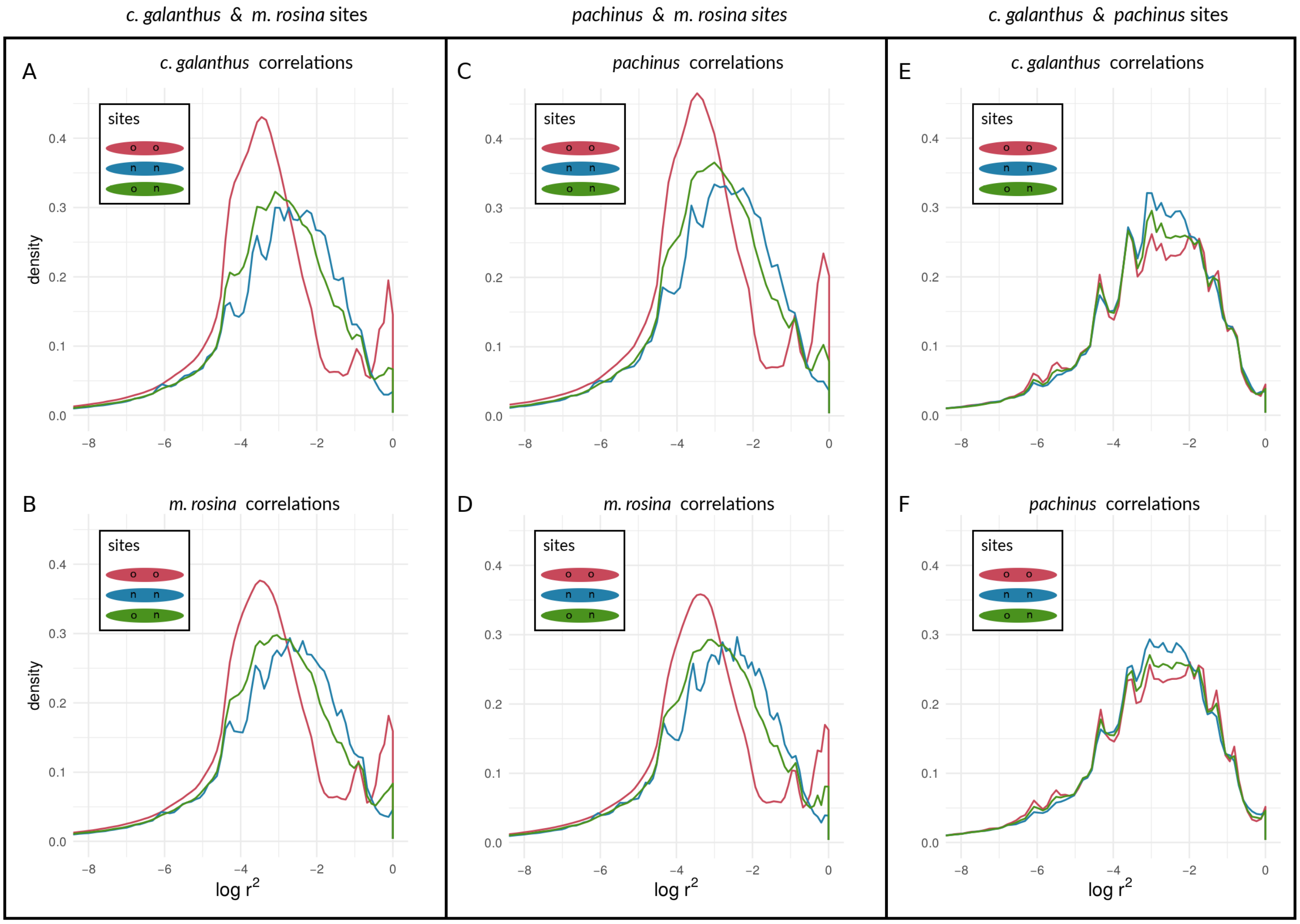

Figure S4: Density curves of within-species LD for loci at different types of sites **on chromosome 18**, determined by F_{ST} outliers. *Log* r^2 values are shown between outliers, between non-outliers and between outliers and non-outliers for species pairs of *H. cydno galanthus* and *H. melpomene rosina* (A & B), *H. pachinus* and *H. melpomene rosina* (C & D), and *H. cydno galanthus* and *H. pachinus* (E & F).

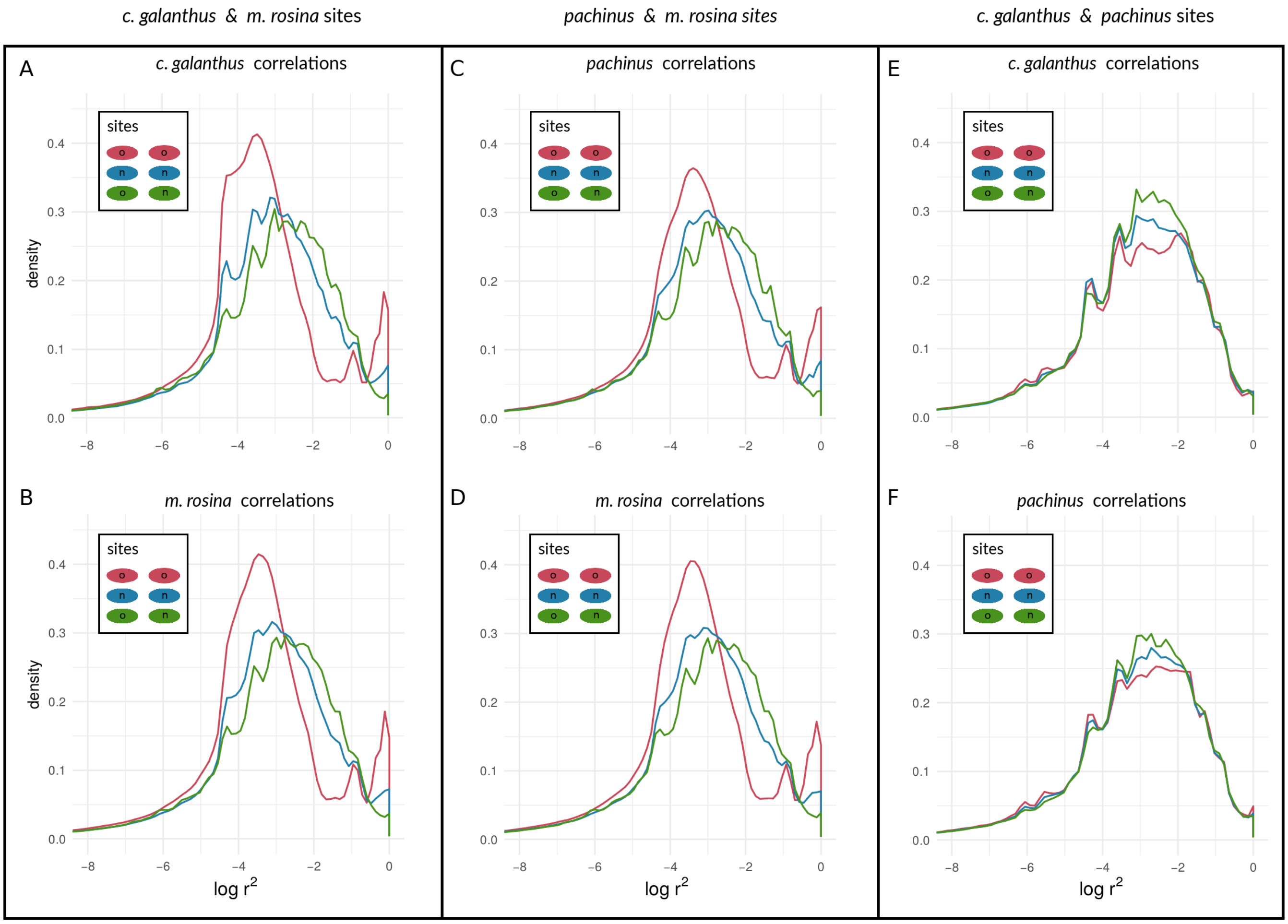

Figure S5: Density curves of within-species LD for loci at different types of sites **on chromosome 2 and respective sites on all other chromosomes**, determined by F_{ST} outliers. *Log* r^2 values are shown between outliers, between non-outliers and between outliers and non-outliers for species pairs of *H. cydno galanthus* and *H. melpomene rosina* (A & B), *H. pachinus* and *H. melpomene rosina* (C & D), and *H. cydno galanthus* and *H. pachinus* (E & F).

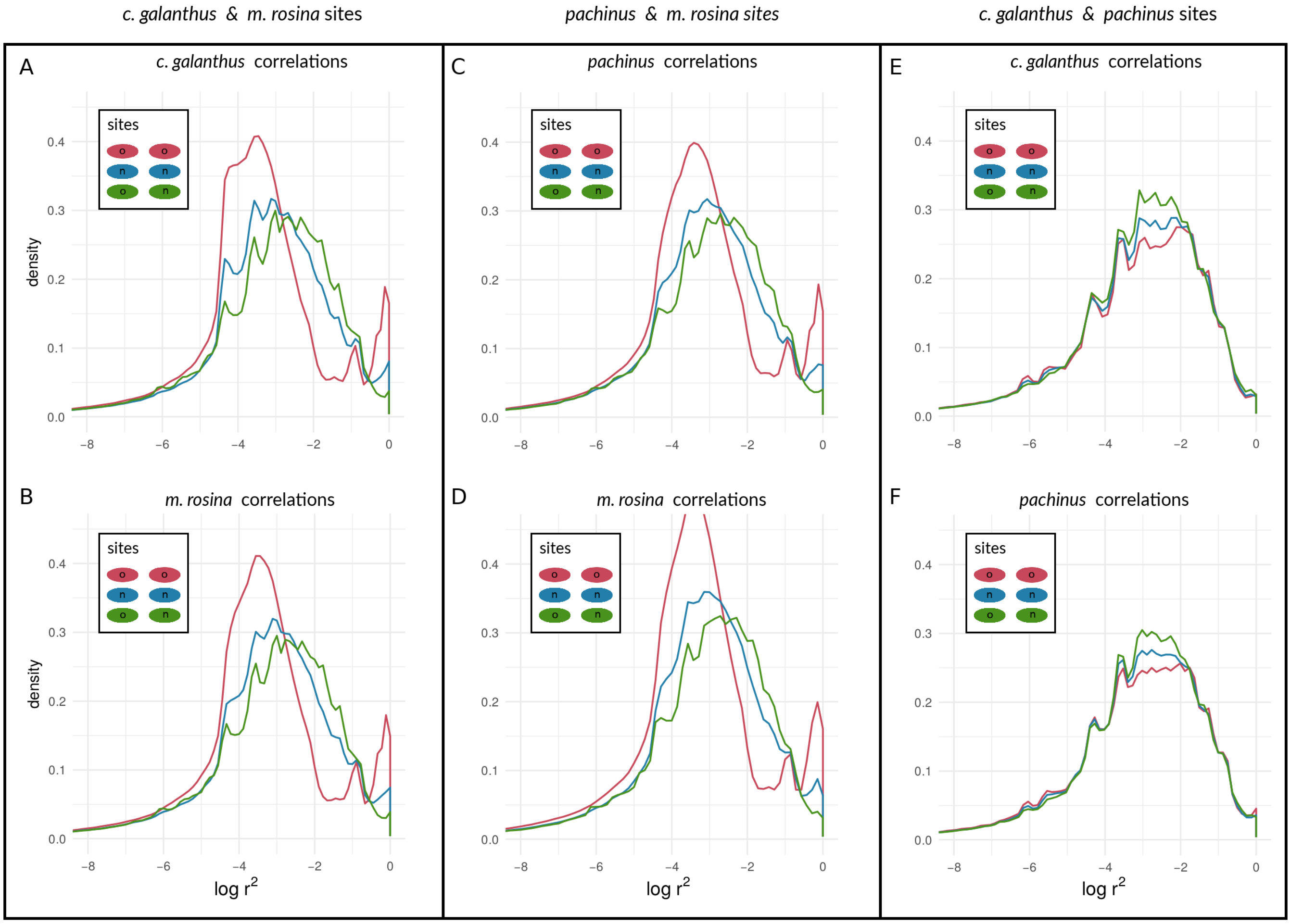

Figure S6: Density curves of within-species LD for loci at different types of sites **on chromosome 7 and respective sites on all other chromosomes**, determined by F_{ST} outliers. *Log* r^2 values are shown between outliers, between non-outliers and between outliers and non-outliers for species pairs of *H. cydno galanthus* and *H. melpomene rosina* (A & B), *H. pachinus* and *H. melpomene rosina* (C & D), and *H. cydno galanthus* and *H. pachinus* (E & F).

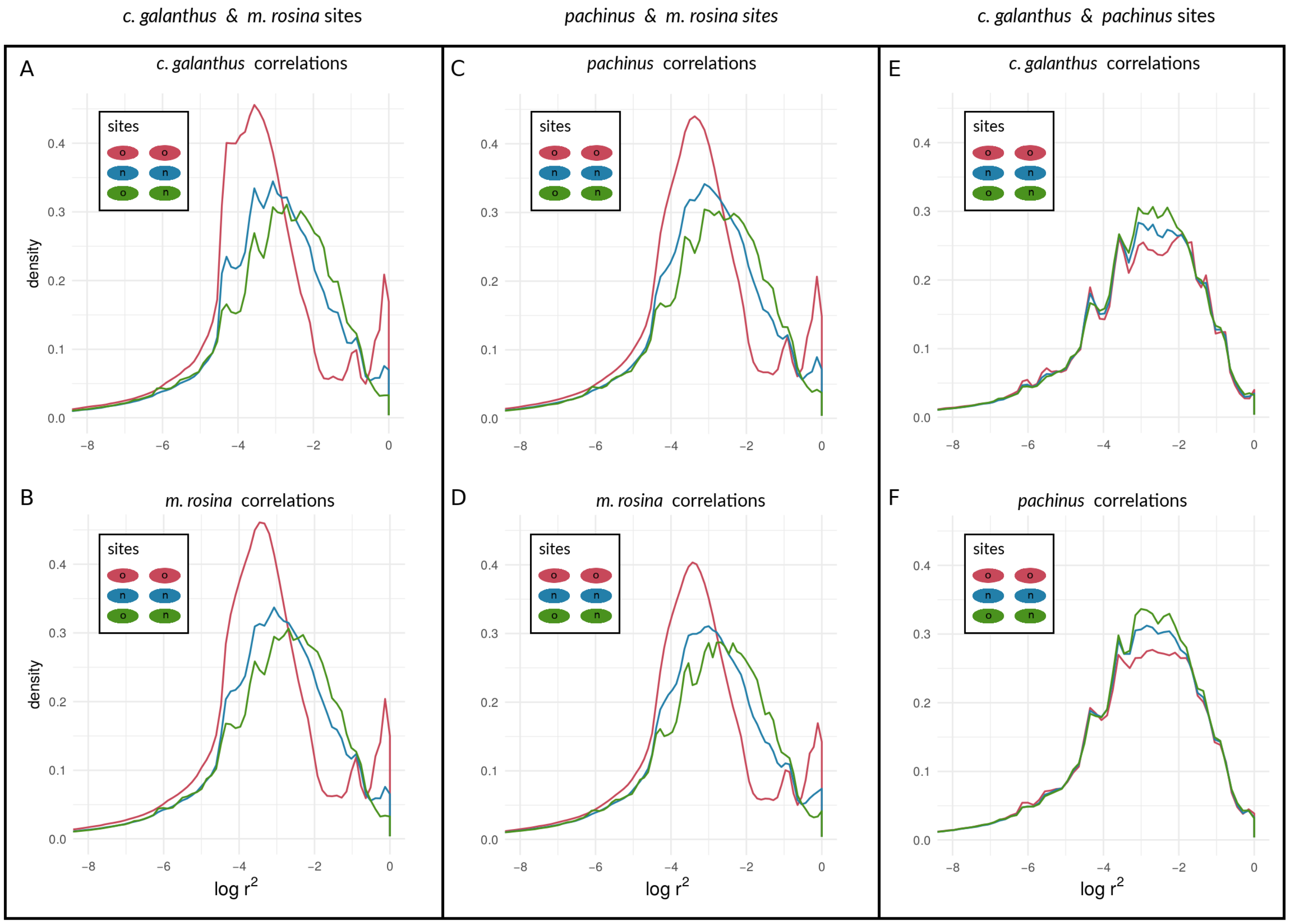

Figure S7: Density curves of within-species LD for loci at different types of sites **on chromosome 10 and respective sites on all other chromosomes**, determined by F_{ST} outliers. *Log* r^2 values are shown between outliers, between non-outliers and between outliers and non-outliers for species pairs of *H. cydno galanthus* and *H. melpomene rosina* (A & B), *H. pachinus* and *H. melpomene rosina* (C & D), and *H. cydno galanthus* and *H. pachinus* (E & F).

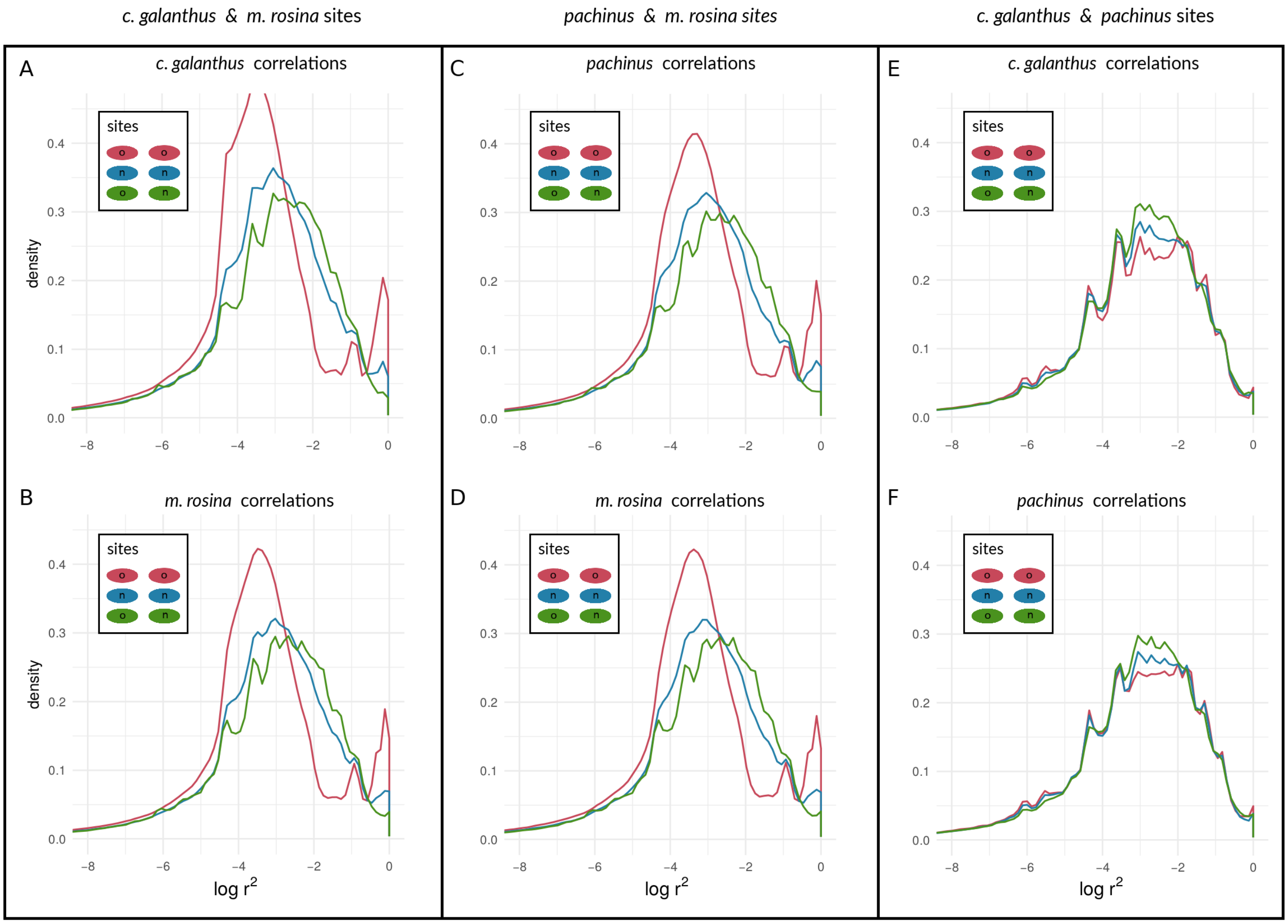

Figure S8: Density curves of within-species LD for loci at different types of sites **on chromosome 18 and respective sites on all other chromosomes**, determined by F_{ST} outliers. *Log* r^2 values are shown between outliers, between non-outliers and between outliers and non-outliers for species pairs of *H. cydno galanthus* and *H. melpomene rosina* (A & B), *H. pachinus* and *H. melpomene rosina* (C & D), and *H. cydno galanthus* and *H. pachinus* (E & F).

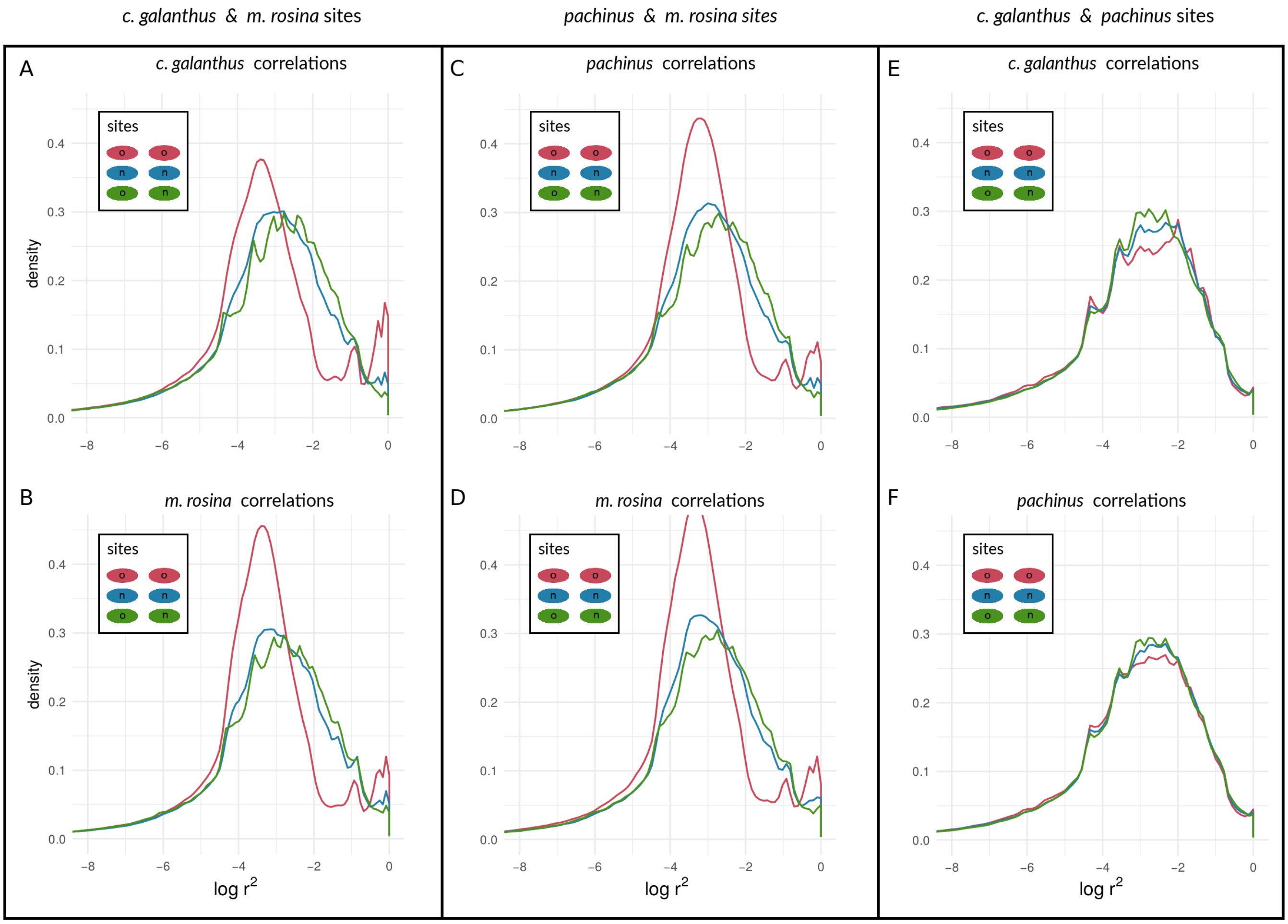

Figure S9: Density curves of within-species LD for loci at different types of sites **on chromosome 21 and respective sites on all other chromosomes**, determined by F_{ST} outliers. *Log* r^2 values are shown between outliers, between non-outliers and between outliers and non-outliers for species pairs of *H. cydno galanthus* and *H. melpomene rosina* (A & B), *H. pachinus* and *H. melpomene rosina* (C & D), and *H. cydno galanthus* and *H. pachinus* (E & F).

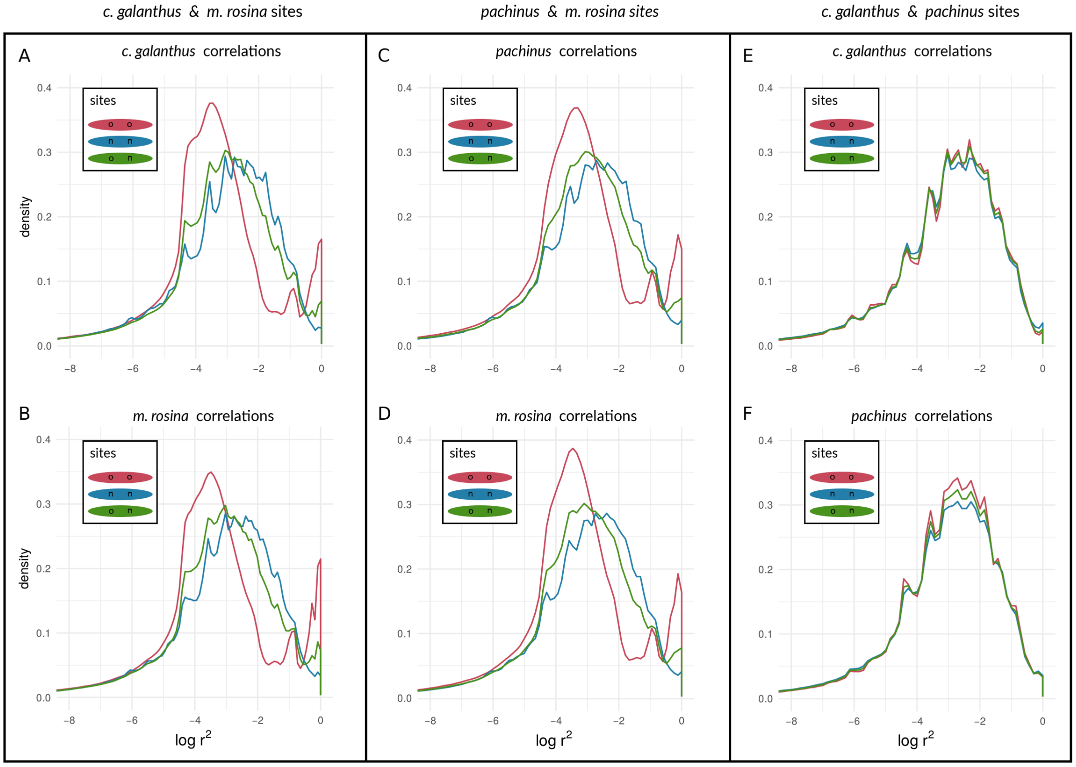

Figure S10: Density curves of within-species linkage disequilibrium for loci at different types of sites (o: outliers; n: non-outliers) on chromosome 2 (representative of autosomes), determined by outliers of allele frequency differences. $\log(r^2)$ values are shown between outliers, between non-outliers, and between outliers and non-outliers for species pairs of *H. cydno galanthus* and *H. melpomene rosina* (A & B), *H. pachinus* and *H. melpomene rosina* (C & D), and *H. cydno galanthus* and *H. pachinus* (E & F).

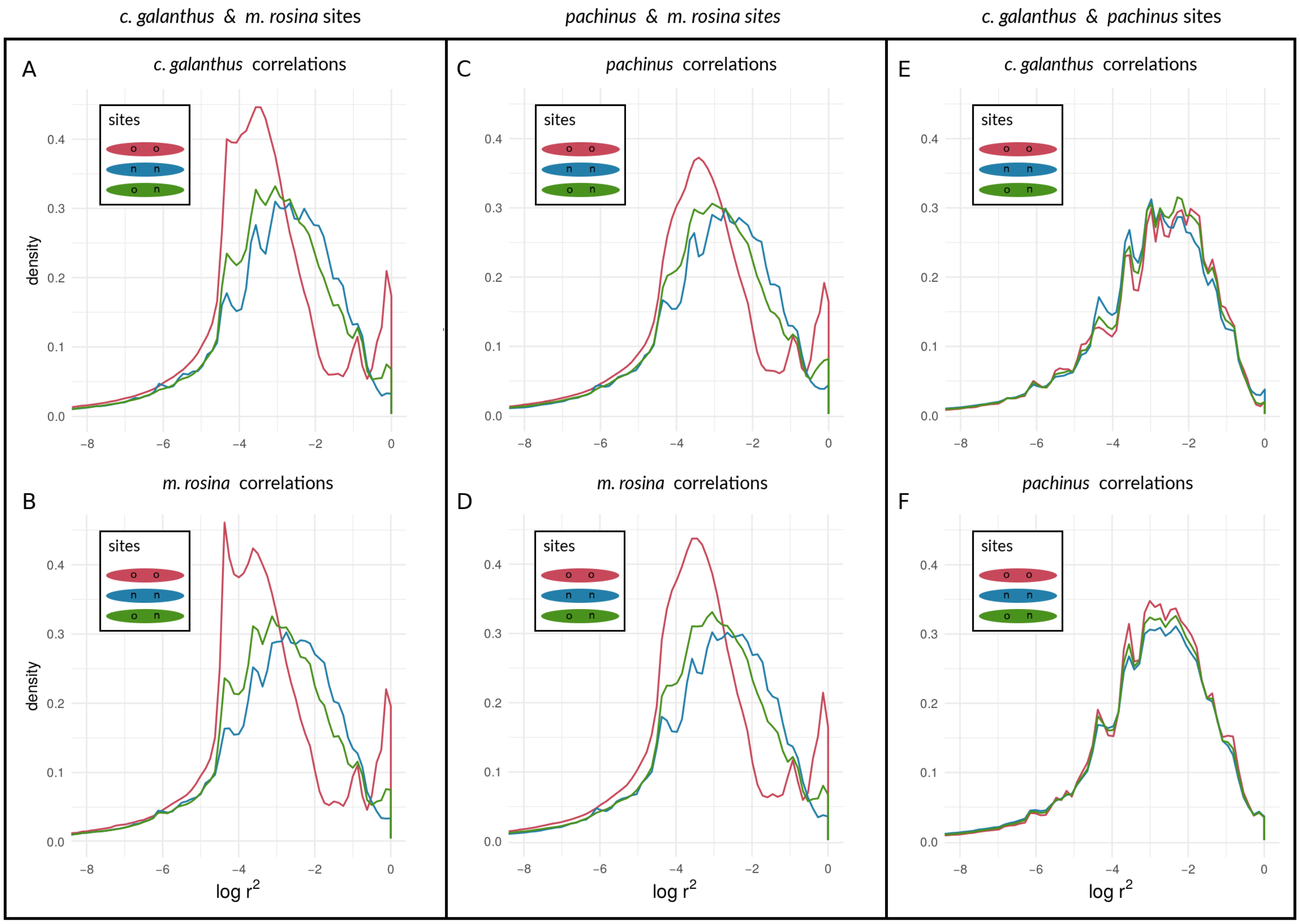

Figure S11: Density curves of within-species LD for loci at different types of sites **on chromosome 7**, determined by outliers of **AFDs**. *Log* r^2 values are shown between outliers, between non-outliers and between outliers and non-outliers for species pairs of *H. cydno galanthus* and *H. melpomene rosina* (A & B), *H. pachinus* and *H. melpomene rosina* (C & D), and *H. cydno galanthus* and *H. pachinus* (E & F).

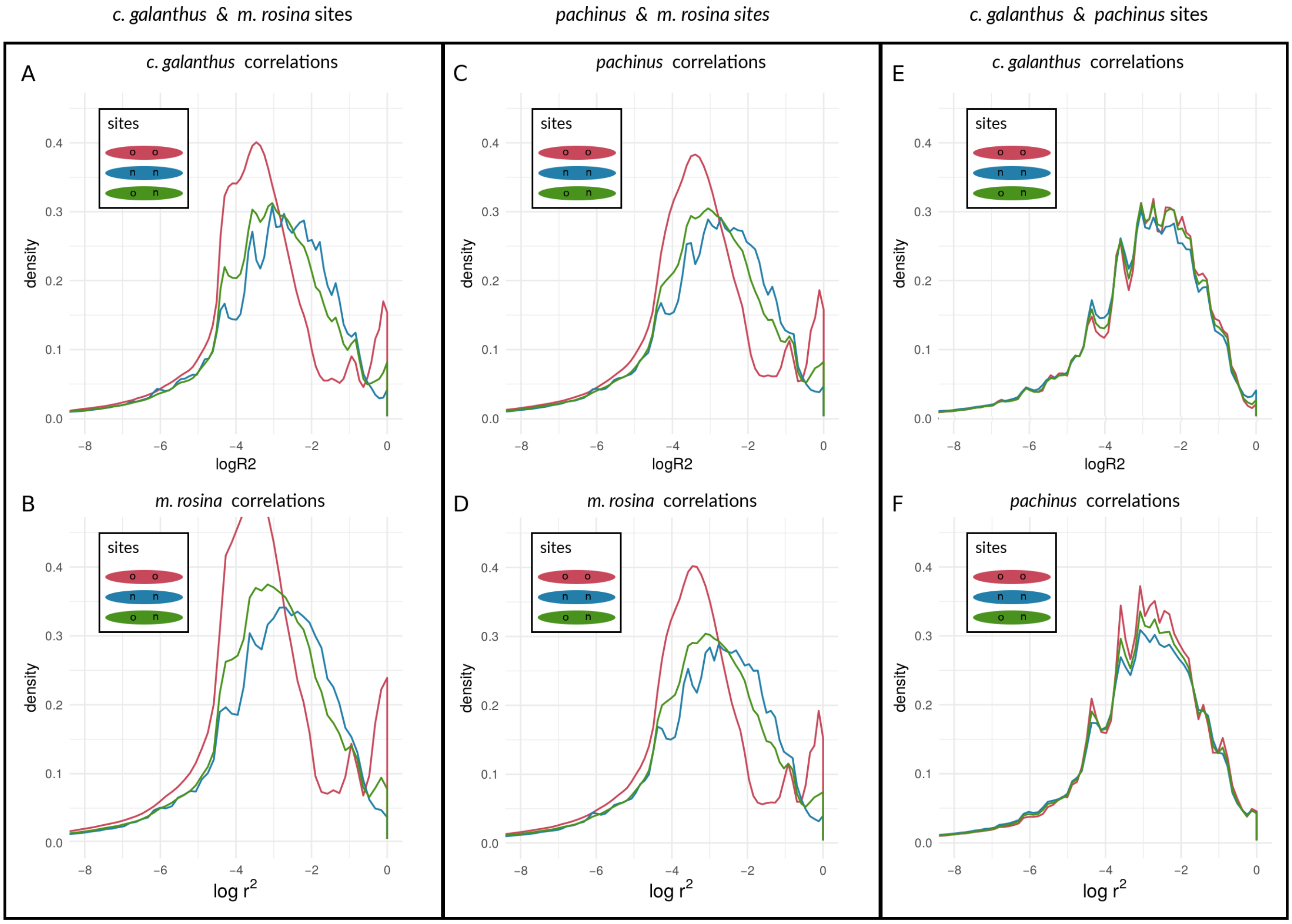

Figure S12: Density curves of within-species LD for loci at different types of sites **on chromosome 10**, determined by outliers of **AFDs**. *Log* r^2 values are shown between outliers, between non-outliers and between outliers and non-outliers for species pairs of *H. cydno galanthus* and *H. melpomene rosina* (A & B), *H. pachinus* and *H. melpomene rosina* (C & D), and *H. cydno galanthus* and *H. pachinus* (E & F).

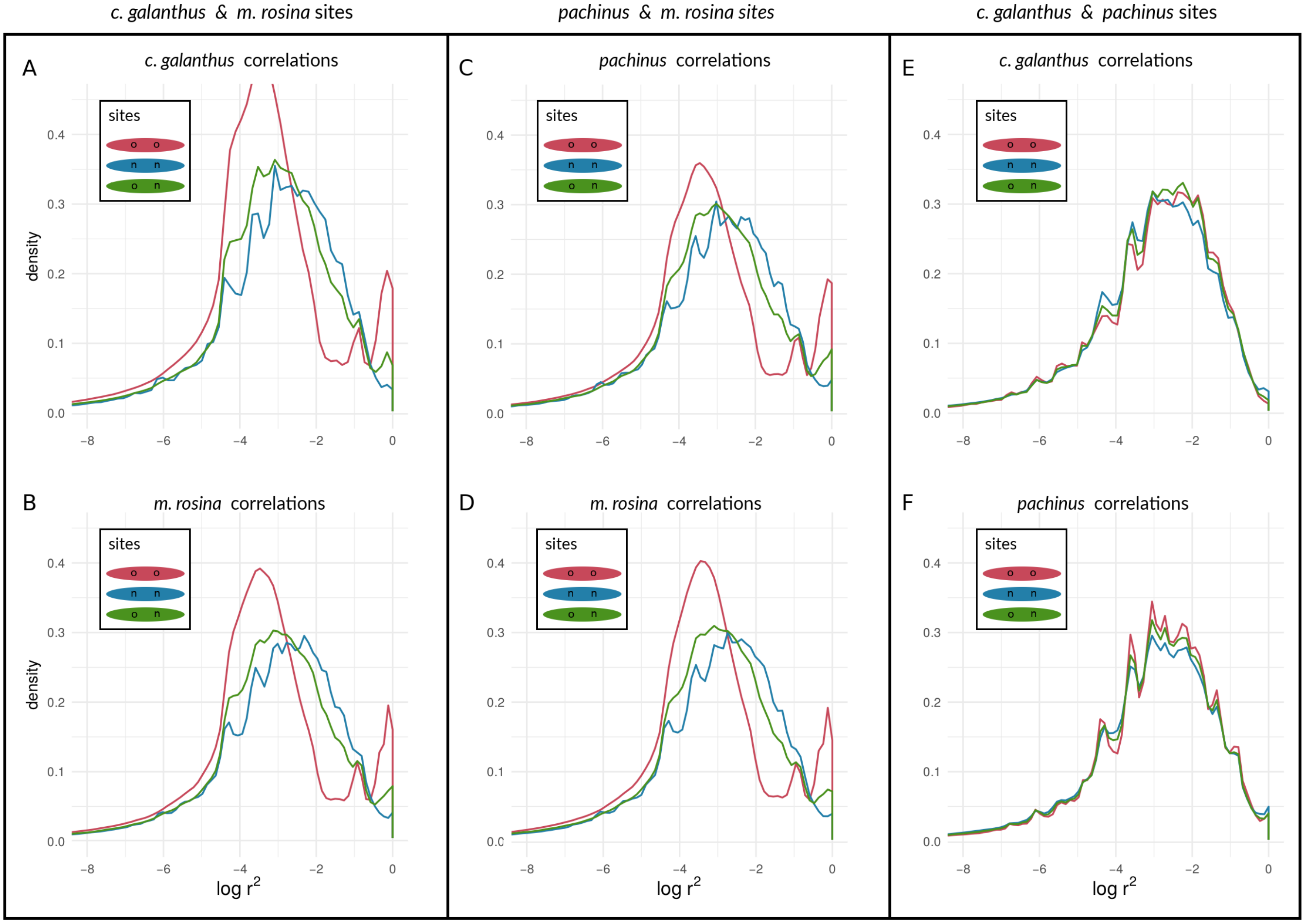

Figure S13: Density curves of within-species LD for loci at different types of sites **on chromosome 18**, determined by outliers of **AFDs**. *Log* r^2 values are shown between outliers, between non-outliers and between outliers and non-outliers for species pairs of *H. cydno galanthus* and *H. melpomene rosina* (A & B), *H. pachinus* and *H. melpomene rosina* (C & D), and *H. cydno galanthus* and *H. pachinus* (E & F).

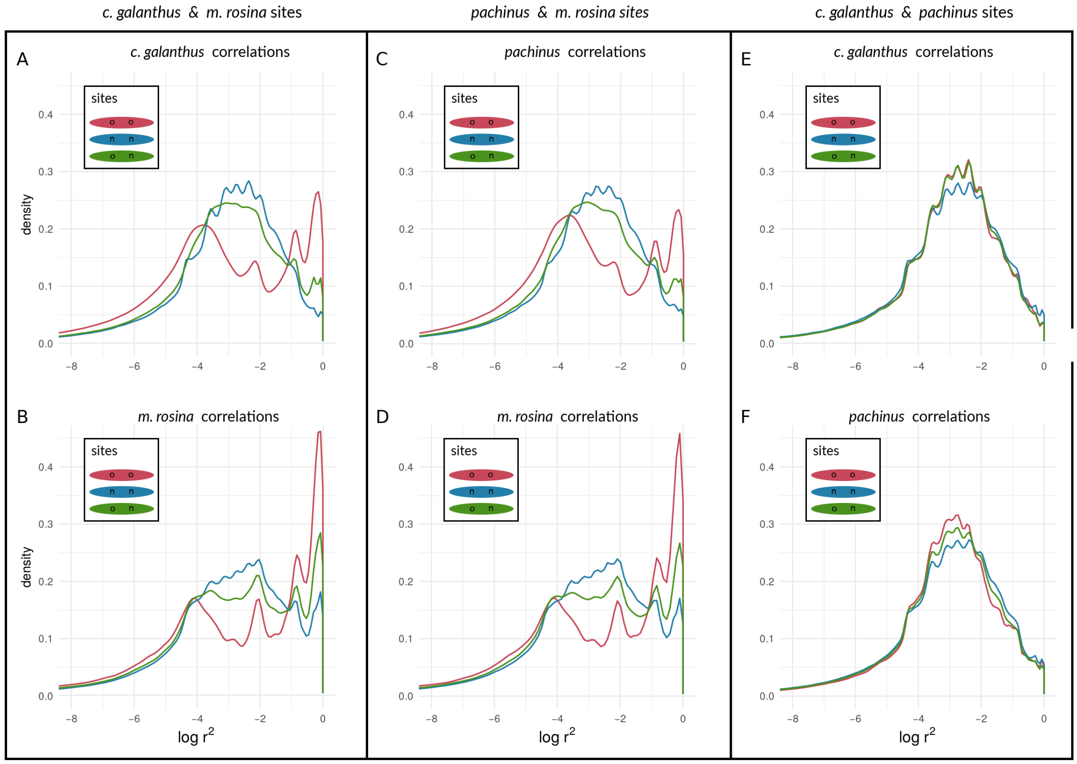

Figure S14: Density curves of within-species linkage disequilibrium for loci at different types of sites (o: outliers; n: non-outliers) on chromosome 21, determined by outliers of allele frequency differences. $\log(r^2)$ values are shown between outliers, between non-outliers, and between outliers and non-outliers for species pairs of *H. cydno galanthus* and *H. melpomene rosina* (A & B), *H. pachinus* and *H. melpomene rosina* (C & D), and *H. cydno galanthus* and *H. pachinus* (E & F).

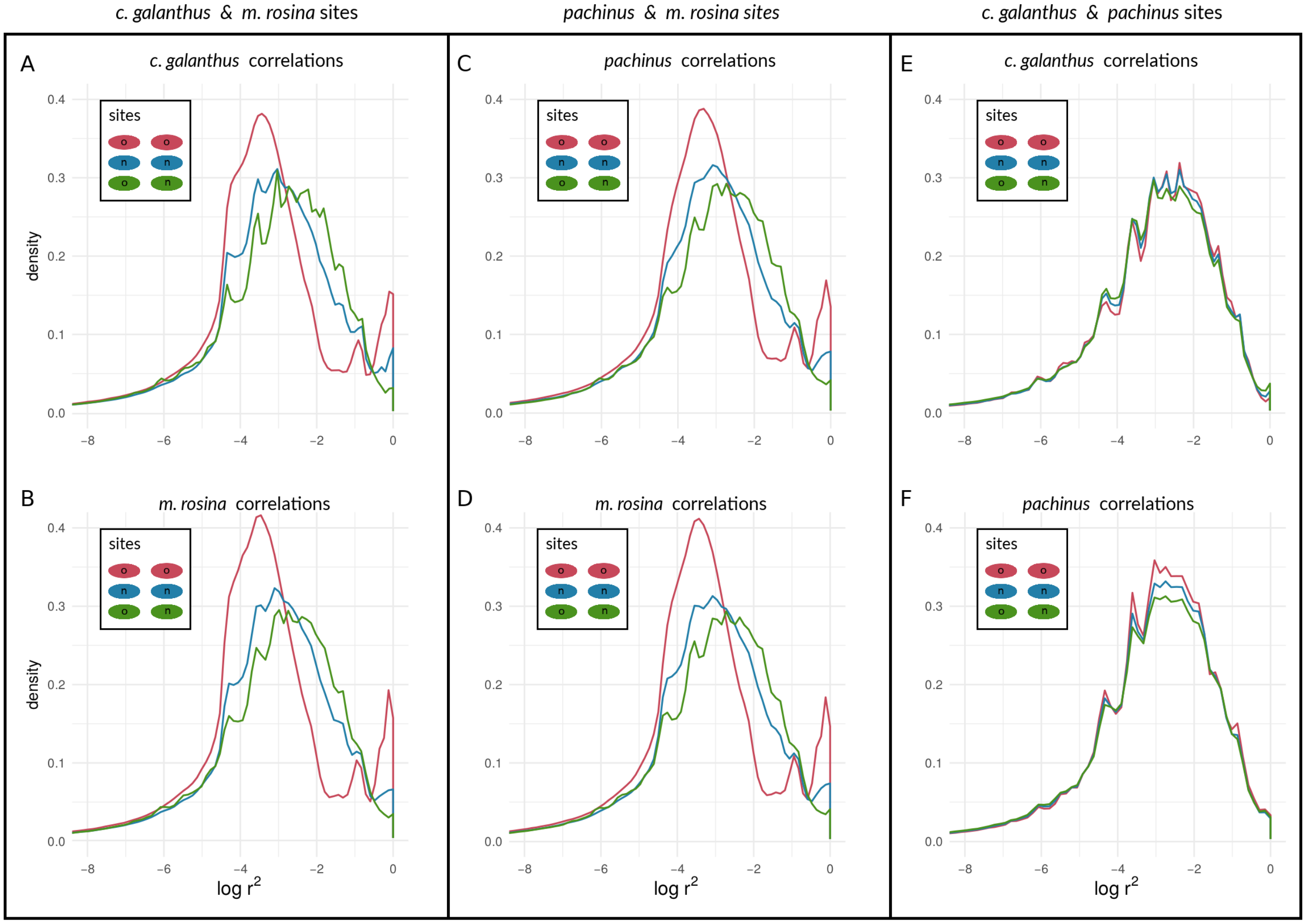

Figure S15: Density curves of within-species LD for loci at different types of sites **on chromosome 2 and respective sites on all other chromosomes**, determined by outliers of **AFDs**. *Log* r^2 values are shown between outliers, between non-outliers and between outliers and non-outliers for species pairs of *H. cydno galanthus* and *H. melpomene rosina* (A & B), *H. pachinus* and *H. melpomene rosina* (C & D), and *H. cydno galanthus* and *H. pachinus* (E & F).

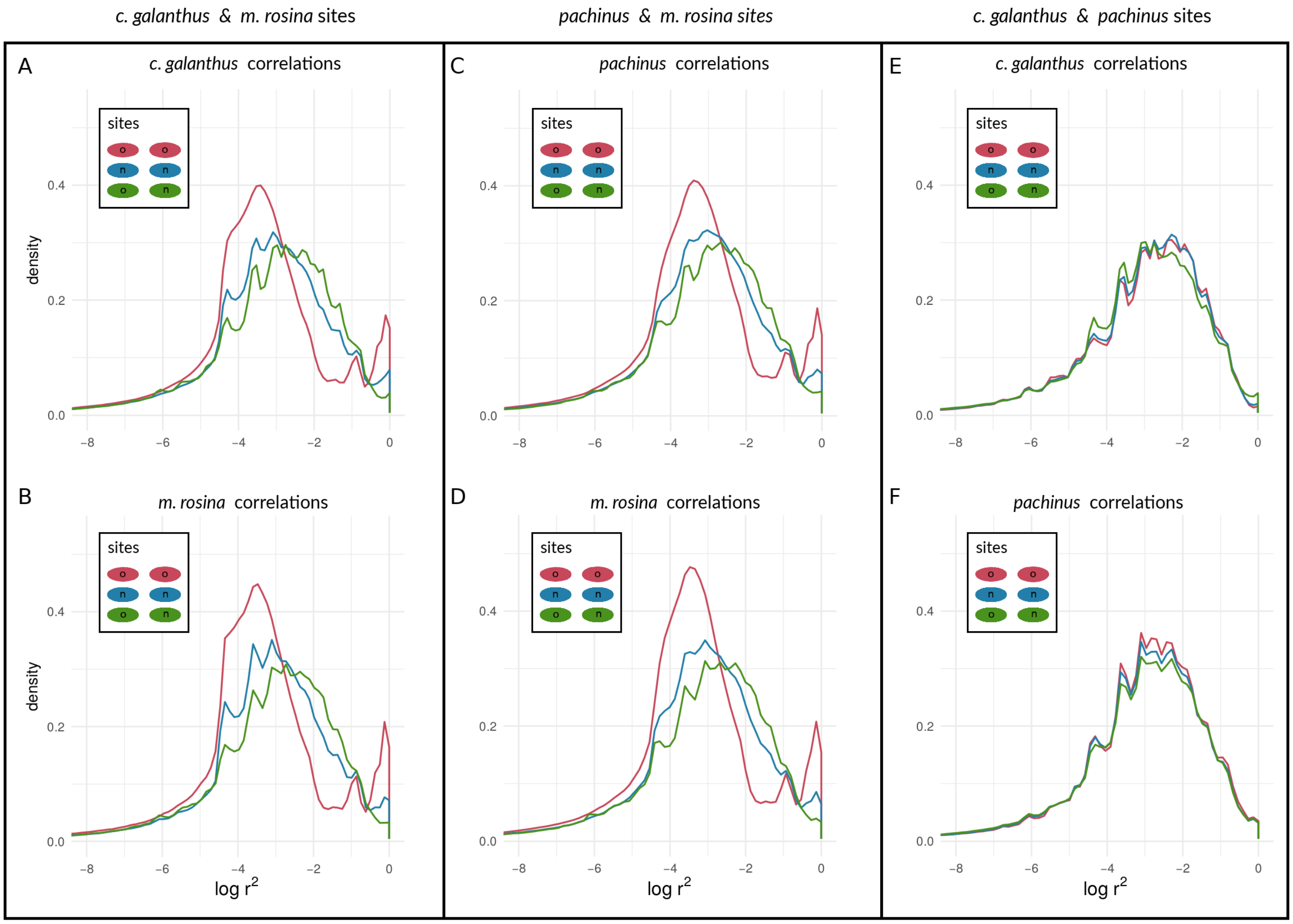

Figure S16: Density curves of within-species LD for loci at different types of sites **on chromosome 7 and respective sites on all other chromosomes**, determined by outliers of **AFDs**. *Log* r^2 values are shown between outliers, between non-outliers and between outliers and non-outliers for species pairs of *H. cydno galanthus* and *H. melpomene rosina* (A & B), *H. pachinus* and *H. melpomene rosina* (C & D), and *H. cydno galanthus* and *H. pachinus* (E & F).

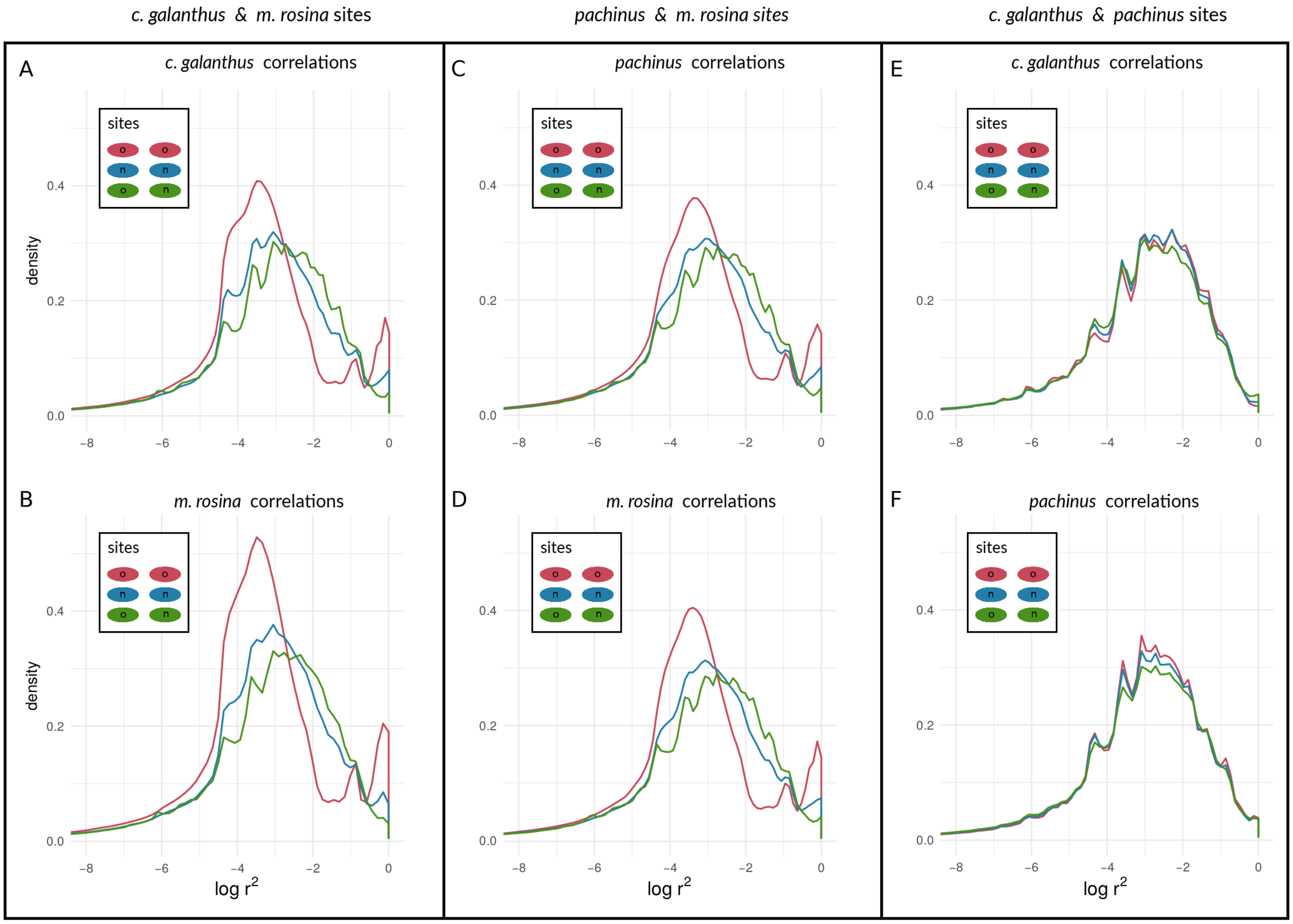

Figure S17: Density curves of within-species LD for loci at different types of sites **on chromosome 10 and respective sites on all other chromosomes**, determined by outliers of **AFDs**. *Log* r^2 values are shown between outliers, between non-outliers and between outliers and non-outliers for species pairs of *H. cydno galanthus* and *H. melpomene rosina* (A & B), *H. pachinus* and *H. melpomene rosina* (C & D), and *H. cydno galanthus* and *H. pachinus* (E & F).

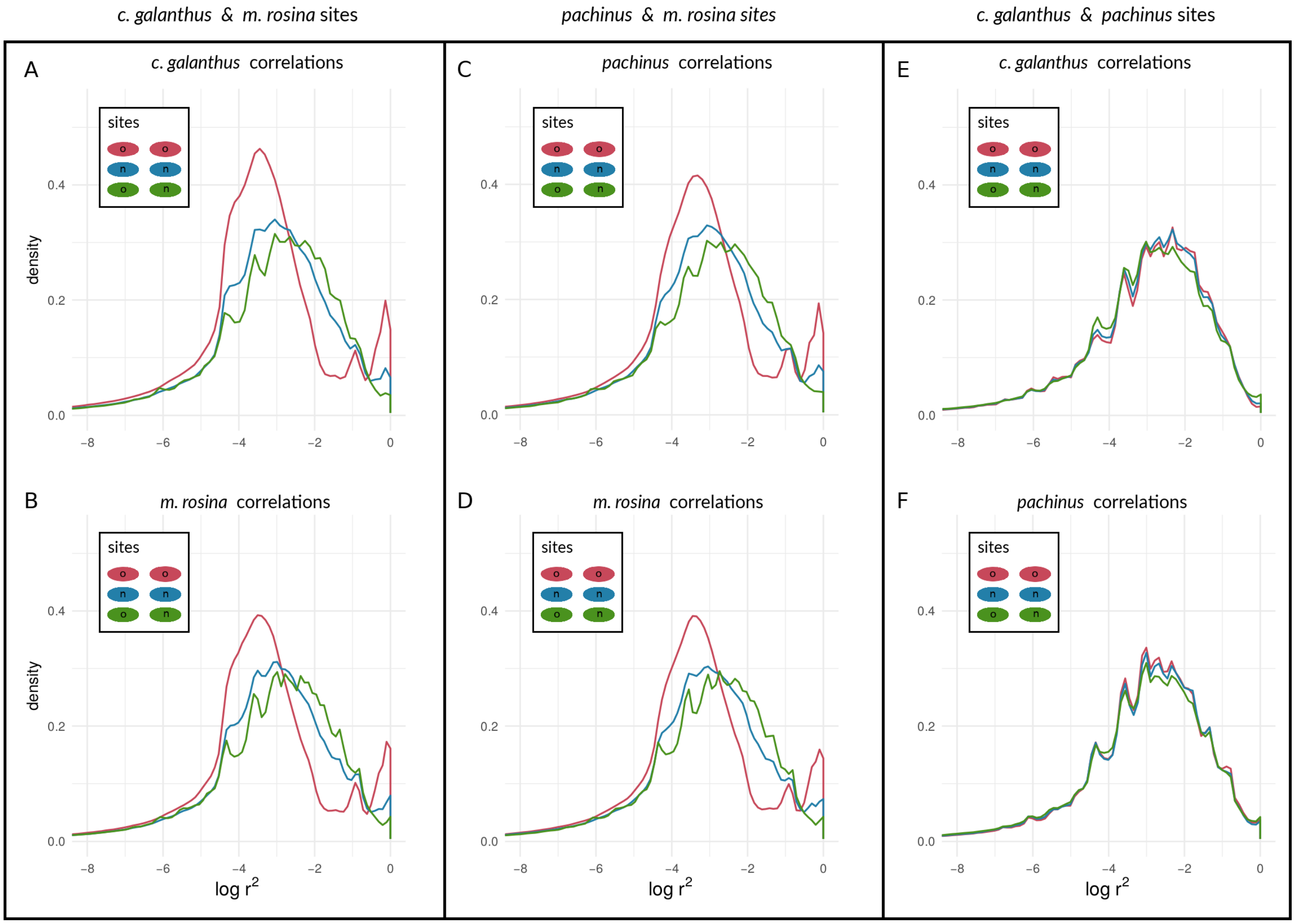

Figure S18: Density curves of within-species LD for loci at different types of sites **on chromosome 18 and respective sites on all other chromosomes**, determined by outliers of **AFDs**. *Log* r^2 values are shown between outliers, between non-outliers and between outliers and non-outliers for species pairs of *H. cydno galanthus* and *H. melpomene rosina* (A & B), *H. pachinus* and *H. melpomene rosina* (C & D), and *H. cydno galanthus* and *H. pachinus* (E & F).

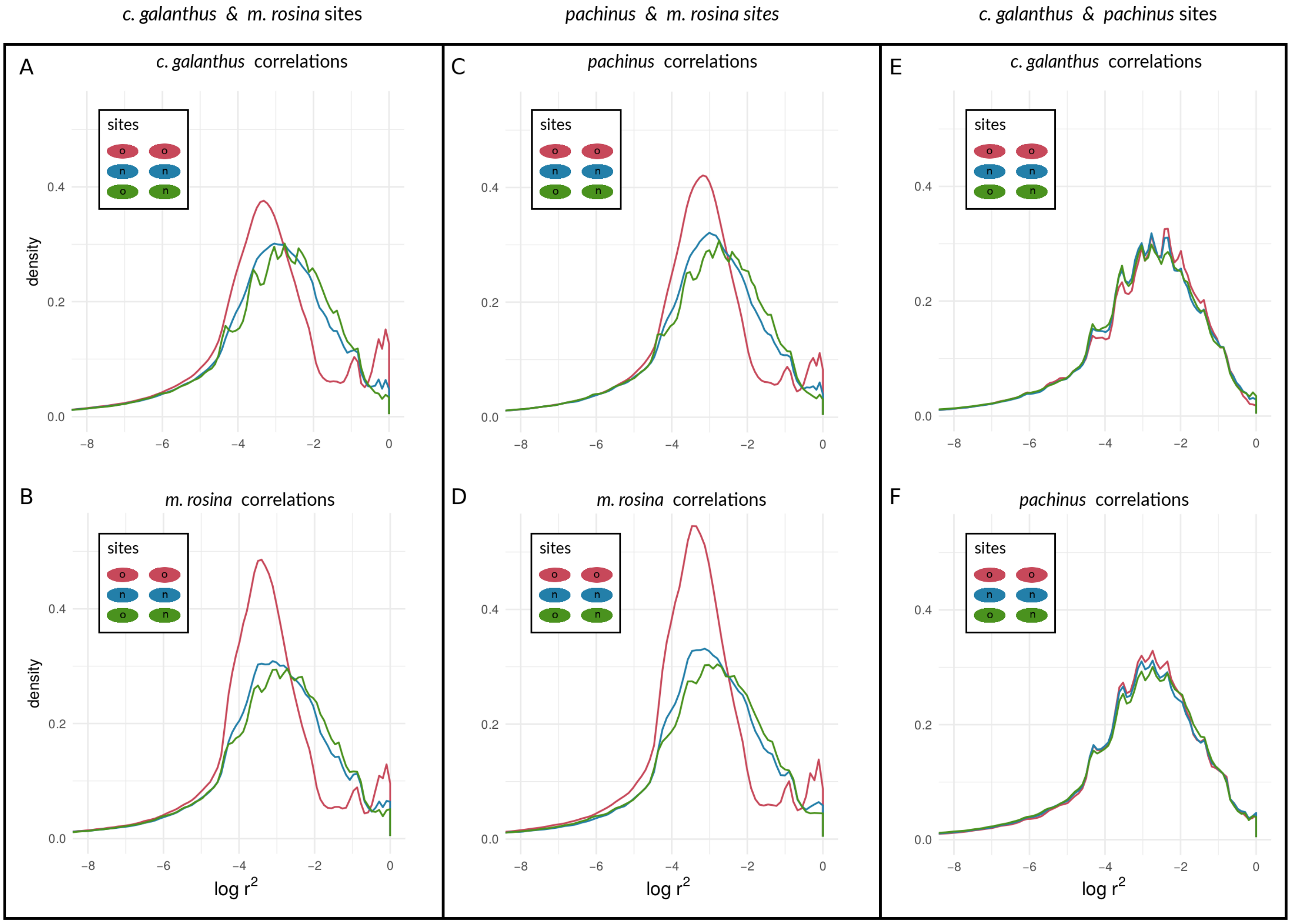

Figure S19: Density curves of within-species LD for loci at different types of sites **on chromosome 21 and respective sites on all other chromosomes**, determined by outliers of **AFDs**. *Log* r^2 values are shown between outliers, between non-outliers and between outliers and non-outliers for species pairs of *H. cydno galanthus* and *H. melpomene rosina* (A & B), *H. pachinus* and *H. melpomene rosina* (C & D), and *H. cydno galanthus* and *H. pachinus* (E & F).
